# TGFB-INHB/activin signaling regulates age-dependent autophagy and cardiac health through inhibition of MTORC2

**DOI:** 10.1080/15548627.2019.1704117

**Published:** 2019-12-29

**Authors:** Kai Chang, Ping Kang, Ying Liu, Kerui Huang, Ting Miao, Antonia P. Sagona, Ioannis P. Nezis, Rolf Bodmer, Karen Ocorr, Hua Bai

**Affiliations:** a Department of Genetics, Development, and Cell Biology, Iowa State University, Ames, IA, USA; b School of Life Sciences, University of Warwick, Coventry, UK; c Development, Aging, and Regeneration Program, Sanford-Burnham-Prebys Medical Discovery Institute, La Jolla, CA, USA

**Keywords:** Atg8a, autophagic flux, dawdle, INHB/activin ligand, semi-automatic optical heartbeat analysis (SOHA), TOR complex 2

## Abstract

Age-related impairment of macroautophagy/autophagy and loss of cardiac tissue homeostasis contribute significantly to cardiovascular diseases later in life. MTOR (mechanistic target of rapamycin kinase) signaling is the most well-known regulator of autophagy, cellular homeostasis, and longevity. The MTOR signaling consists of two structurally and functionally distinct multiprotein complexes, MTORC1 and MTORC2. While MTORC1 is well characterized but the role of MTORC2 in aging and autophagy remains poorly understood. Here we identified TGFB-INHB/activin signaling as a novel upstream regulator of MTORC2 to control autophagy and cardiac health during aging. Using *Drosophila* heart as a model system, we show that cardiac-specific knockdown of TGFB-INHB/activin-like protein daw induces autophagy and alleviates age-related heart dysfunction, including cardiac arrhythmias and bradycardia. Interestingly, the downregulation of *daw* activates TORC2 signaling to regulate cardiac autophagy. Activation of TORC2 alone through overexpressing its subunit protein rictor promotes autophagic flux and preserves cardiac function with aging. In contrast, activation of TORC1 does not block autophagy induction in *daw* knockdown flies. Lastly, either *daw* knockdown or *rictor* overexpression in fly hearts prolongs lifespan, suggesting that manipulation of these pathways in the heart has systemic effects on longevity control. Thus, our studies discover the TGFB-INHB/activin-mediated inhibition of TORC2 as a novel mechanism for age-dependent decreases in autophagic activity and cardiac health.

**Abbreviations:** AI: arrhythmia index; BafA1: bafilomycin A_1_; BMP: bone morphogenetic protein; CQ: chloroquine; CVD: cardiovascular diseases; DI: diastolic interval; ER: endoplasmic reticulum; HP: heart period; HR: heart rate; MTOR: mechanistic target of rapamycin kinase; NGS: normal goat serum; PBST: PBS with 0.1% Triton X-100; PDPK1: 3-phosphoinositide dependent protein kinase 1; RICTOR: RPTOR independent companion of MTOR complex 2; ROI: region of interest; ROUT: robust regression and outlier removal; ROS: reactive oxygen species; R-SMAD: receptor-activated SMAD; SI: systolic interval; SOHA: semi-automatic optical heartbeat analysis; TGFB: transformation growth factor beta; TSC1: TSC complex subunit 1

## Introduction

Aging is associated with an exponential increase in the incidence of cardiovascular diseases (CVD) [1,2]. Age-related changes in cardiovascular structure and output have been linked to increased risk of coronary heart disease, sudden cardiac death and stroke in the elderly population [3]. During normal aging, the left ventricular wall of human hearts becomes thickened and the diastolic filling rate of left ventricle gradually decreases, while the left ventricular systolic performance at rest remains no change with age [3]. Several mechanisms underlying these age-associated changes in cardiovascular structure and function have been proposed, for example, changes in growth factor signaling, decreased cellular quality control, altered calcium handling, elevated extracellular matrix deposition or fibrosis, increased mitochondria damage, and the production of reactive oxygen species (ROS) [2]. Resolving the contributing mechanisms underlying the age-dependent decline of cardiovascular function is critical for the development of therapeutic interventions for the treatment of cardiovascular diseases.

Cellular quality control systems, such as macroautophagy (hereinafter referred to as autophagy), are essential to the maintenance of tissue homeostasis during aging [4]. Autophagy is a highly conserved process that is responsible for the degradation and recycling of damaged organelles, protein aggregates, and other cytoplasmic substances [5]. It is generally accepted that autophagy activity declines with age [6]. However, how autophagic activity is altered in aging hearts remains unclear. Disruption of autophagy pathways often leads to loss of tissue homeostasis and tissue dysfunction. For example, knocking out autophagy gene *Atg5* in the mouse heart accelerates cardiac aging, including an increase in left ventricular hypertrophy and decrease in fractional shortening [7]. As a key autophagy regulator, MTOR (mechanistic target of rapamycin kinase) plays an important role in the regulation of cardiac tissue homeostasis [2]. The MTOR signaling consists of two structurally and functionally distinct protein complexes, MTORC1 and MTORC2 [8]. Rapamycin-mediated suppression of MTORC1 induces autophagy and protects mouse cardiomyocytes from oxidative stress [9]. *Drosophila* genome contains evolutionarily conserved Tor kinase and several major TOR complex subunits (e.g., raptor and Lst8 for TORC1, rictor, Sin1 and Lst8 for TORC2) [10]. *Drosophila* MTORC1 promotes electrical pacing-induced heart failure [11], and high-fat-diet-induced cardiomyopathy [12]. Unlike MTORC1, the role of MTORC2 in autophagy, tissue homeostasis, and cardiac aging remains poorly understood. Two recent studies show that disruption of MTORC2 subunit RICTOR (RPTOR independent companion of MTOR complex 2) induces cardiac dysfunction in mice [13,14]. Interestingly, 2 MTOR complexes exhibit distinct regulation on lifespan. Inhibition of MTORC1 is known to prolong lifespan [15], while deletion of RICTOR, the key component of MTORC2, shortens lifespan of male mice [16]. However, the molecular basis for MTORC2-regulated aging and tissue homeostasis remains to be further examined.

TGFB (transforming growth factor beta) is an evolutionarily conserved signaling pathway that regulates a wide variety of cellular processes, such as proliferation, differentiation, apoptosis, inflammation, and fibrosis [17]. Recent studies demonstrate that TGFB factors promote cardiac fibrosis during aging [18], while reduction of TGFB signaling improves cardiac health in aged mice through the regulation of microRNA *MIR29* [19]. As in mammals, TGFB family in *Drosophila* has 2 major branches, BMP (bone morphogenetic protein) and TGFB-INHB/activin signaling pathways [17]. In both TGFB pathways, signaling starts with ligand binding to a receptor complex composed of type I and type II receptor kinases, followed by phosphorylation of receptor-activated SMAD (R-SMAD). Recently, we and other groups show that TGFB-INHB/activin signaling regulates muscle proteostasis and longevity in *Drosophila* [20,21]. However, it remains to be determined whether TGFB family proteins, in particular, INHB/activin, regulate cardiac homeostasis and function.

While investigating the role of *Drosophila* TGFB-INHB/activin signaling in the regulation of autophagy and age-related cardiomyopathy, we find that TGFB-INHB/activin signaling genetically interacts with TORC2, but not TORC1 to control age-dependent decreases in autophagy and cardiac health. We further show that the activation of TORC2 alone can induce autophagy and preserve cardiac function during aging. Thus, our studies uncover a novel crosstalk between TGFB-INHB/activin signaling and TORC2 that is essential for the maintenance of autophagic activity, tissue homeostasis, and cardiac health with aging.

## Results

### Heart-specific knockdown of TGFB-INHB/activin-like ligand daw slows cardiac aging

**Figure 1.**
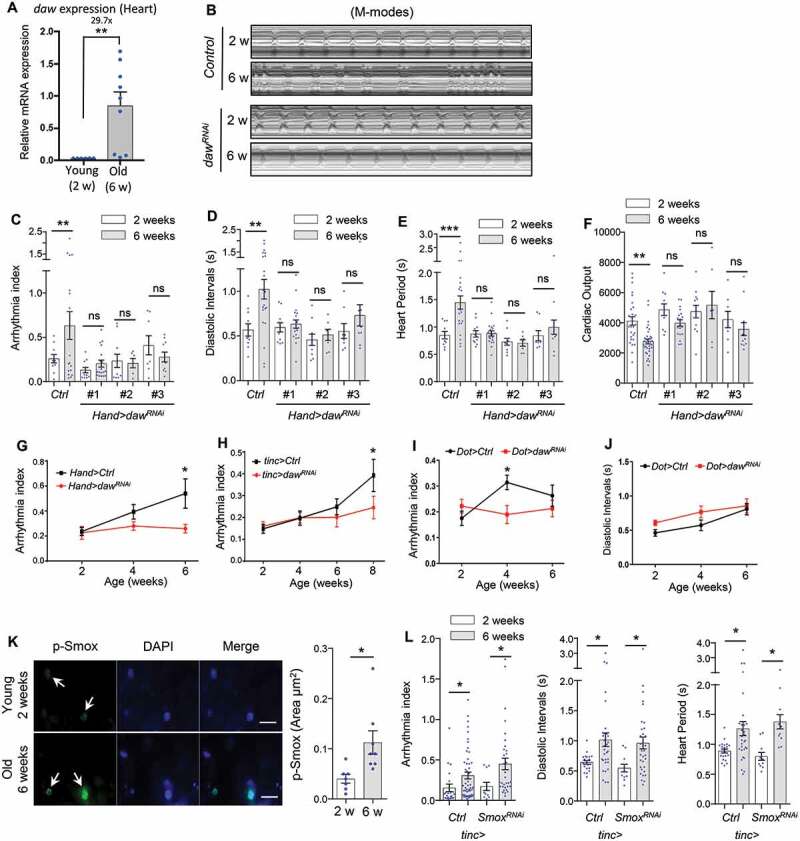
Heart-specific knockdown of *daw* slows cardiac aging. (A) QRT-PCR analysis of *daw* expression in young and old fly hearts. N = 3. Student t-test (** p < 0.01). (B) Representative M-mode traces (8 s) showing age-dependent movement of heart wall in control and heart-specific *daw* knockdown flies (*Hand-gal4> UAS-daw^RNAi^*). (C-F) Age-related changes in arrhythmia index, diastolic intervals, heart period, and cardiac output in control (*Ctrl*) and cardiac-specific *daw* knockdown flies (*daw^RNAi^*). Flies were cultured at 40% relative humidity. *Hand-gal4* driver was used to knockdown gene expression specifically in cardiac tissues (cardiomyocytes and pericardial cells). Results from three independent *UAS-daw^RNAi^* lines are shown (RNAi #1: BDSC, 34974, RNAi #2: Vienna Drosophila Resource Center, 105309, RNAi #3: BDSC, 50911). N = 7 ~ 20. Two-way ANOVA followed by Tukey multiple comparisons test (* p < 0.05, ** p < 0.01, *** p < 0.001, ns = not significant). The interaction between genotype and age is statistically significant for heart period (*p* = 0.0041) and diastolic interval (*p* = 0.0243). (G-I) Age-dependent changes in cardiac arrhythmia between control and *daw* knockdown using various tissue drivers, *Hand-gal4* (cardiomyocytes and pericardial cells), *tinc-gal4* (cardiomyocytes), and *Dot-gal4* (pericardial cells). N = 25 ~ 31. Student t-test (* p < 0.05, ** p < 0.01, *** p < 0.001). (J) Age-dependent changes in diastolic intervals between control and *daw* knockdown in pericardial cells (*Dot-gal4*). N = 13 ~ 26. Student t-test (* p < 0.05, ** p < 0.01, *** p < 0.001). (K) Immunostaining of p-Smox in fly hearts at young (2 weeks) and old ages (6 weeks). Arrows indicate cardiomyocyte nuclei and positive p-Smox staining. Scale bar: 20 μm. Quantification shown on the right. N = 8. Student t-test (* p < 0.05). Data are represented as mean ± SEM in all figures. (L) Age-dependent changes in arrhythmia index, diastolic intervals, heart period between control and *Smox* knockdown (*tinc-gal4*). N = 11 ~ 31. Student t-test (* p < 0.05).

Our previous study demonstrated that the TGFB-INHB/activin-like protein daw regulates tissue homeostasis (especially in indirect flight muscle) and longevity in *Drosophila* [20]. Interestingly, we found that the mRNA expression of *daw* was significantly upregulated in aging fly hearts (29.7-fold) (Figure 1A), suggesting that TGFB-INHB/activin signaling may play a role in regulating cardiac aging. To investigate this possibility, we knocked down the expression of *daw* specifically in the heart with a binary GAL4/UAS system where we crossed *UAS-daw* RNAi lines into a cardiac-specific tissue driver (*Hand-gal4 or Hand4.2-gal4*) (Figure S1A,B). We analyzed cardiac performance in young and old flies using a high-speed video imaging system (semi-automatic optical heartbeat analysis, SOHA) [22]. M-Mode traces from the SOHA analysis indicated that knockdown of *daw* preserved cardiac contractility at advanced ages (Figure 1B). In wild-type flies, aged hearts showed increased arrhythmia (Figures 1C, S1I and S1J, data from three wild-type genotypes), lengthened diastolic intervals and heart period (bradycardia, slow heart rate) (Figure 1D,E), and decreased cardiac output (Figure 1F). Systolic intervals normally remain unchanged (Figure S1E). There is a small age-related reduction in fractional shortening (Figure S1F), but the diastolic and systolic diameters were not significantly altered (Figure S1G,H). Interestingly, cardiac-specific knockdown of *daw* attenuated the age-dependent increase in arrhythmia, diastolic intervals, and heart period (Figure 1C–E). Knockdown of *daw* also maintained relatively normal cardiac output at advanced ages (Figure 1F). Similar results were observed from three independent *daw* RNAi lines (the knockdown efficiency of *daw* RNAi was verified by qRT-PCR previously [20], as well as reconfirmed in the present study) (Figure S1C,D). However, cardiac-specific overexpression of *daw* did not lead to premature cardiac aging phenotypes (Figure S1K), which may be because the ectopic expression of *daw* in the heart did not generate active daw proteins.

The *Hand-gal4* line drives gene expression in both cardiomyocytes and pericardial cells (Figure S1A). To test the cardiomyocyte-specific role of daw in cardiac aging, we then crossed *daw* RNAi into a cardiomyocyte-specific driver *tinc-gal4 (or tincΔ4-gal4*) (Figure S1A). Similar to the results from *Hand-gal4* (Figure 1C,G), cardiomyocyte-specific knockdown of *daw* prevented the age-related increase in cardiac arrhythmia (Figure 1H). We also tested whether *daw* expression in pericardial cells can regulate cardiac aging using a pericardial cell-specific driver *Ugt36A1/Dot-gal4*. Interestingly, knockdown of *daw* in pericardial cells also alleviated age-induced arrhythmia (Figure 1I), but not diastolic interval (Figure 1J). Thus, reduction of daw signaling can alleviate age-related cardiac dysfunction, particularly the age-induced arrhythmia, through both cardiomyocyte-specific regulation and non-autonomous signaling from pericardial cells.

It is known that TGFB-INHB/activin signaling typically functions through its downstream transcription factor SMAD2 (or Smox in *Drosophila*). We noticed that during aging the phosphorylation of Smox/SMAD2 was elevated in the fly heart nuclei (Figure 1K). To test the role of Smox in cardiac aging, we performed SOHA analysis on flies with cardiac-specific *Smox* knockdown. Surprisingly, knockdown of *Smox* in the heart did not attenuate the age-dependent increase in arrhythmia, diastolic intervals, and heart period (Figure 1L). These findings suggest that daw may regulate cardiac aging through a Smox-independent pathway (e.g., MAPK/p38 signaling [23]).

### Cardiomyocyte-specific knockdown of INHB/activin receptor *babo* delays cardiac aging

**Figure 2.**
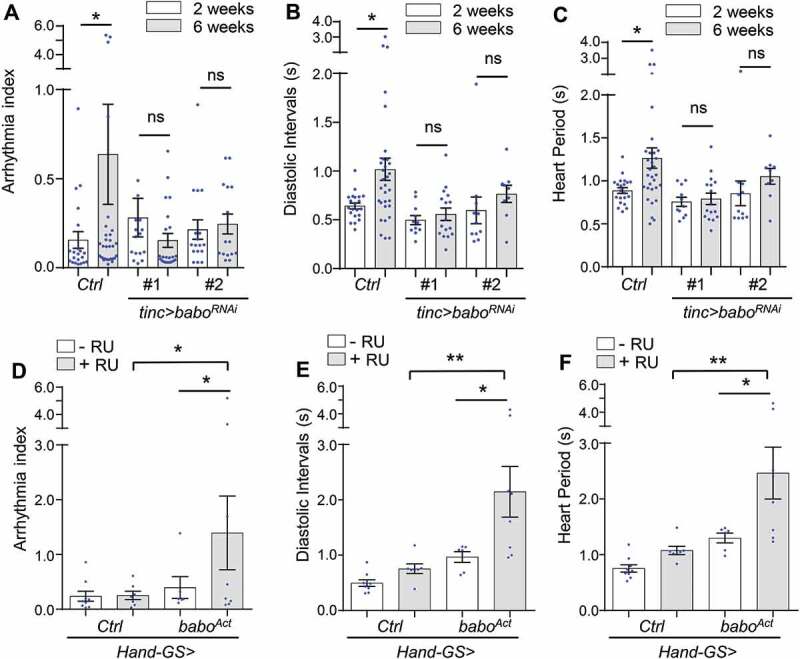
Cardiomyocyte-specific knockdown of INHB/activin receptor *babo* delays cardiac aging. (A-C) Age-dependent changes in cardiac arrhythmia, diastolic intervals, and heart period in control (*Ctrl*) and cardiomyocyte-specific *babo* knockdown flies (*babo ^RNAi^*). Flies were cultured at 40% relative humidity. *tinc-gal4* driver was used. Results from two independent *UAS-babo^RNAi^* lines are shown (RNAi #1: BDSC, 25933, RNAi #2: BDSC, 40866). N = 15 ~ 30. One-way ANOVA (*** p < 0.001, ** p < 0.01, * p < 0.05, ns = not significant). (D-F) Changes in cardiac arrhythmia, diastolic intervals, and heart period in fly hearts expressing constitutively activated *babo* (*babo^Act^*). GeneSwitch heart driver *Hand-GS-gal4* was used to induce adult-onset *babo* activation. RU: RU486 (Mifepristone). Flies were cultured at 40% relative humidity. N = 6 ~ 9. One-way ANOVA (*** p < 0.001, ** p < 0.01, * p < 0.05, ns = not significant).

In *Drosophila*, INHB/activin-like ligand daw signals through the type I receptor babo, the fly homolog of mammalian ACVR1B/ALK4 (activin A receptor, type 1B) [17]. To investigate whether daw acts through its receptor babo to modulate cardiac aging, we first tested if babo plays a similar role in preventing the age-dependent increase in cardiomyopathy. Consistent with the results from *daw* knockdown, we found that cardiomyocyte-specific knockdown of *babo* blocked the age-related increase in cardiac arrhythmia, diastolic intervals, and heart period (Figure 2A–C). Two independent *babo* RNAi lines were used and both gave similar results (the knockdown efficiency of *babo* RNAi was verified by qRT-PCR in our previous study [20]. Knockdown of *babo* did not affect age-dependent changes in cardiac output (Figure S1L), which may be attributed to the similar diastolic diameter and systolic diameter between wild-type and *babo* knockdown flies (Figure S1M,N). These results suggest that babo and daw regulate both common and distinct aspects of cardiac function.

In a reciprocal experiment, we used a GeneSwitch heart driver (*Hand-GS-gal4*) to express a constitutively active form of *babo* (*babo^Act^, or babo^Q302D^*) specifically in adult hearts. GeneSwitch heart driver was used because constitutively expressing active *babo* in the heart (*Hand>babo^Act^*) led to severe developmental arrest at pupal stages (data not shown). We found that cardiac expression of activated *babo* significantly increased cardiac arrhythmia, diastolic intervals, and heart period already at young ages (Figure 2D–F). Taken together, these results suggest that INHB/activin type I receptor babo plays an important role in age-related cardiomyopathy, especially cardiac arrhythmia, diastolic function, and heart rate.

### daw negatively regulates autophagy in fly hearts

**Figure 3.**
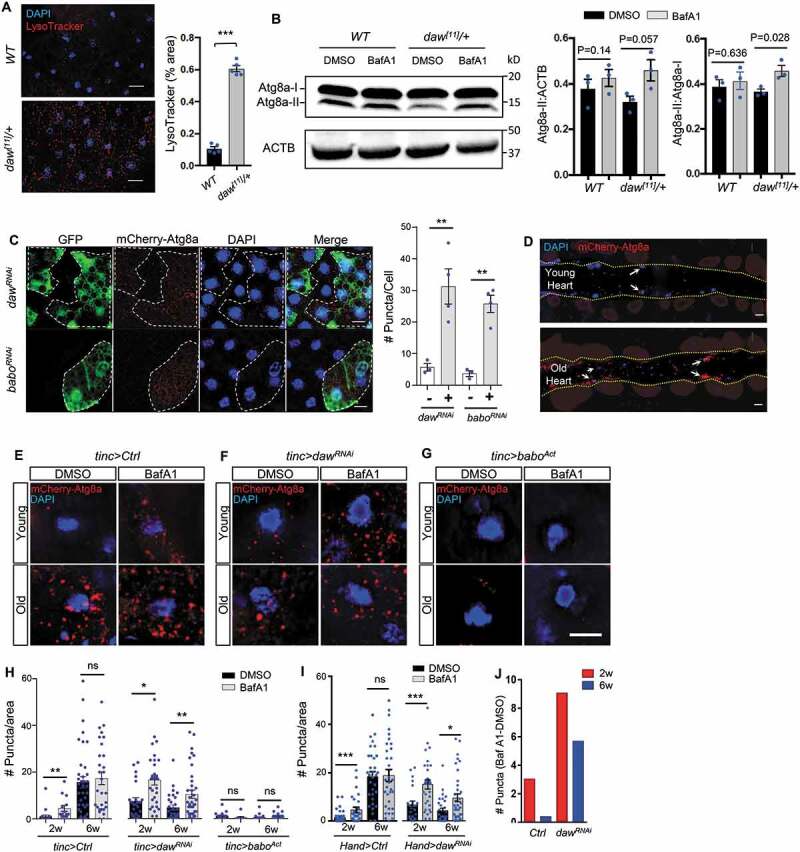
Daw negatively regulates autophagy in fly hearts. (A) Representative images of LysoTracker staining of young adult fat body between wild-type (*WT*) and heterozygous *daw^[11]^/+* mutants. Scale bar: 20 μm. Quantification shown on the right. N = 5. Student t-test (*** p < 0.001). (B) Western blot analysis on Atg8a lipidation between *WT* and *daw^[11]^/+* mutants. GABARAP antibodies were used to detect Atg8a. The lower band represents lipidated Atg8a (Atg8a-II), and the upper band represents non-lipidated Atg8a (Atg8a-I). Abdominal fly carcass is incubated with 5 μM of BafA1 prior to western blots. ACTB (actin beta) is used as the loading control. Quantification of band intensity shown on the right. N = 3. Student t-test (*** p < 0.001). (C) Representative images of mosaic analysis on autophagosome staining in larval fat body. Fat body clones were generated by crossing *daw^RNAi^* and *babo^RNAi^* lines into a FLPout line carrying mCherry-Atg8a reporter (*hs-flp; endogenous P-3xmCherry-Atg8a, UAS-GFP/Cyo; Act>CD2> Gal4,UAS-Dcr-2*). RNAi Clones are GFP-positive cells (dashed lines). Scale bar: 20 μm. Quantification of autophagosome puncta shown on the right. N = 5. Student t-test (** p < 0.01). (D) Representative images of fly hearts expressing autophagosome reporter mCherry-Atg8a at both young and old ages. Arrows indicate cardiomyocyte nuclei and surrounding autophagosomes. Abdominal segments A2-A3 are shown. Heart tube is located between two yellow dashed lines. (E-G) Representative images of mCherry-Atg8a reporter surrounding cardiomyocyte nuclei in control, *daw^RNAi^*, and *babo^Act^* flies with or without BafA1 treatment. Semi-intact hearts were incubated with 100 nM of bafilomycin A_1_ (BafA1) for 2 h prior to immunostaining. Scale bar: 10 μm. (H) Quantification of age-dependent changes in autophagic flux in control, *daw^RNAi^*, and *babo^Act^* fly hearts. *tinc-gal4* was used to drive the expression of mCherry-Atg8a reporter and gene knockdown. N = 5 ~ 7. One-way ANOVA (*** p < 0.001, ** p < 0.01, * p < 0.05, ns = not significant). (I) Quantification of age-dependent changes in autophagic flux in control and *daw^RNAi^* fly hearts. *Hand -gal4* was used to drive the expression of mCherry-Atg8a reporter and *daw* knockdown. N = 5 ~ 7. One-way ANOVA (*** p < 0.001, ** p < 0.01, * p < 0.05, ns = not significant). (J) Quantification of BafA1-induced mCherry-Atg8a-positive puncta between control and *daw^RNAi^*. The data represent the differences in the number of puncta between BafA1 and DMSO treatments.

Autophagy is one of the key mechanisms in maintaining tissue hemostasis, and its activity normally declines with age [4,6,7]. Our previous studies showed that TGFB-INHB/activin signaling regulates autophagosome formation and Atg8a transcription in *Drosophila* flight muscle [20]. It is likely that daw regulates cardiac aging by modulating autophagic activity. To first verify the role of daw in autophagy-lysosome activity, we used LysoTracker staining, a well-established autophagy marker in flies [24], to monitor the lysosomal acidification in adult fat body. Consistent with our previous finding, heterozygous *daw^[11]^* loss-of-function mutants showed increased LysoTracker staining, suggesting an elevated basal autophagy/lysosome activity (Figure 3A). We furthermore showed that mutation of *daw* increased autophagic flux, indicated by the induction of lipidated Atg8a (Atg8a-II) upon the treatment of lysosome inhibitor bafilomycin A_1_ (BafA1) (Figure 3B). Atg8a is the fly homolog of mammalian LC3-GABARAP family proteins. A rabbit monoclonal antibody for human GABARAP (E1J4E) [25 26–27] was used in the lipidation assay. The specificity of the antibody has been previously verified [28] and further confirmed in the present study (Figure S2, also see Methods). Finally, the mosaic analysis revealed that somatic cell clones in larval fat body expressing RNAi against either *daw* or *babo* (marked by GFP) showed increased numbers of autophagosome, indicated by mCherry-Atg8a-positive puncta (Figure 3C). These data suggest that TGFB-INHB/activin signaling negatively regulates autophagy and lysosome activities.

Next, we examined whether daw plays a similar role in modulating autophagy in fly hearts. Immunostaining with GABARAP (E1J4E) antibodies showed that the number of autophagosome in the heart was higher in *daw^[11]^* mutants compared to wild-type (Figure S3A,B). Because autophagy is a dynamic process, the increased number of autophagosome could result from elevated autophagy activities, or blockage of lysosomal degradation [29]. To test these possibilities, we next examined the role of daw in the regulation of autophagic flux (the turnover of autophagosome). We exposed semi-intact fly hearts to BafA1, the V-ATPase inhibitor that blocks both lysosomal acidification and autophagosome-lysosome fusion [30]. We found that BafA1 treatment resulted in a higher induction in the number of Atg8a-positive autophagosome in *daw^[11]^* hearts compared to wild-type hearts (Figure S3A–C). Thus, the results suggest that daw negatively regulates autophagic flux in fly hearts.

### Knockdown of *daw* preserves autophagic activity during cardiac aging

Aging tissues show reduced autophagy and accumulated cellular damages. We next examined whether inhibition of INHB/activin-daw signaling in fly hearts can alleviate age-dependent deregulation of autophagy. Using a mCherry-Atg8a reporter line, we observed a large accumulation of Atg8a-positive autophagosome in aged hearts of wild-type flies (Figure 3D,E,H,I). The age-related accumulation of autophagosome was not due to increased expression of the cardiac driver. Because both *Hand-gal4* and *tinc-gal4* drivers showed either reduced or no change of expression during aging (Figure S1A,B). In fact, the accumulation of autophagosome is likely due to the blockage of autophagosome turnover (autophagic flux), because BafA1 treatment induced Atg8a-positive puncta in young hearts, but not in the old hearts (Figure 3E,H,I,J). To confirm this finding, we monitored autophagosome using an alternative method, the immunostaining with GABARAP antibody (Figure S4A,B). We observed a similar accumulation of autophagosome in aged hearts, and the autophagic flux was also reduced during aging (Figure S4A,B). Furthermore, we found that the receptor protein ref(2)P/SQSTM1/p62, a marker for defective aggrephagy [31], was accumulated in aging hearts and Atg8a mutants (Figure S5), suggesting an age-related impairment in autophagy/lysosome degradation system. We also tested autophagic flux using mCherry-GFP-Atg8a tandem reporter. However, we hardly observed any GFP signals in fly hearts, even after BafA1 treatment (Figure S4C). This is quite different from fat body tissue, where BafA1 treatment significantly induced GFP signals when the tandem reporter was expressed using *r4-gal4* (Figure S4D).

Intriguingly, flies with heart-specific *daw* knockdown maintained high levels of cardiac autophagosome turnover (or active autophagic flux) at both young and old ages, as indicated by the induction of autophagosome number upon BafA1 treatment (Figure 3F,H,I,J). Notably, constitutively activated babo suppressed the autophagic flux in both young and old hearts (Figure 3G,H). Activation of babo also blocked the age-dependent accumulation of autophagosome in old fly hearts (Figure 3G,H), which suggests that besides the regulation of autophagosome turnover (degradation), TGFB-INHB/activin signaling also plays an important role in autophagosome initiation and formation. Taken together, our findings suggest that reduction of INHB/activin-daw signaling promotes autophagosome turnover and preserves autophagic activity during cardiac aging.

### Inhibition of autophagy, but not activation of TORC1, blocks *daw* knockdown-mediated cardioprotection

**Figure 4.**
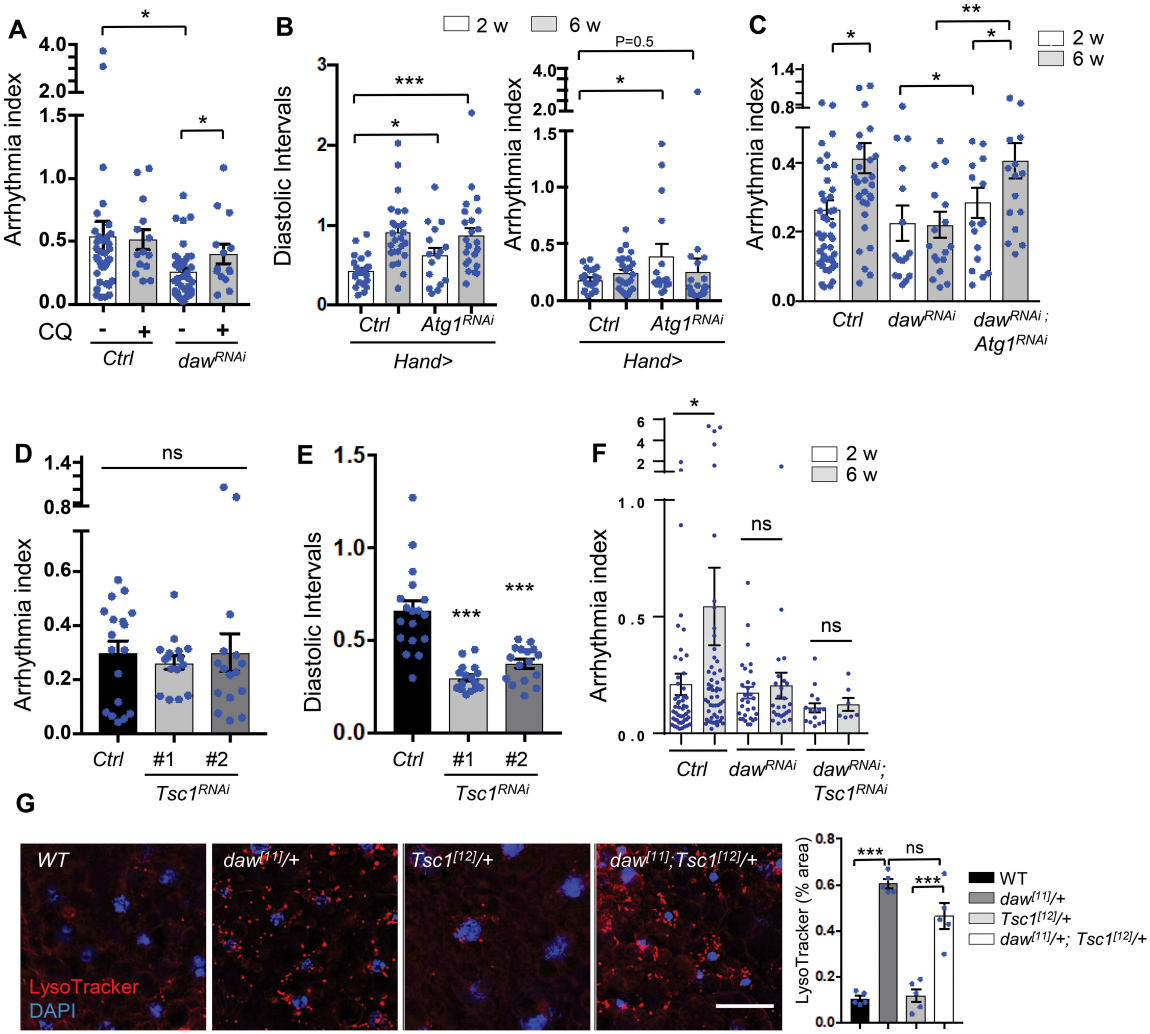
Inhibition of autophagy, but not activation of TORC1, blocks *daw* knockdown-mediated cardioprotection. (A) Cardiac arrhythmia of chloroquine-treated (20 mM, 24 h) 6-week-old control and *daw* knockdown flies (*Hand-gal4* used). N = 14 ~ 38. One-way ANOVA (*** p < 0.001, ** p < 0.01, * p < 0.05, ns = not significant). (B) Cardiac diastolic intervals and arrhythmia in heart-specific knockdown of *Atg1* at young and old ages (*Hand-gal4* used). N = 16 ~ 19. One-way ANOVA (*** p < 0.001, * p < 0.05). (C) Age-dependent changes in cardiac arrhythmia in control, *daw^RNAi^*, and *daw^RNAi^; Atg1^RNAi^* flies (*tinc-gal4* used). N = 16 ~ 35. One-way ANOVA (*** p < 0.001, ** p < 0.01, * p < 0.05, ns = not significant). (D-E) Cardiac arrhythmia and diastolic intervals of *Tsc1* knockdown flies (*tinc-gal4* used). Two independent *Tsc1* RNAi lines were used (RNAi-1: BDSC, 52931, RNAi-2: BDSC, 54034). N = 16 ~ 18. One-way ANOVA (*** p < 0.001, ** p < 0.01, * p < 0.05, ns = not significant). (F) Age-dependent changes in cardiac arrhythmia in control, *daw^RNAi^*, and *daw^RNAi^; Tsc1^RNAi^* flies (*tinc-gal4* used). N = 7 ~ 30. One-way ANOVA (*** p < 0.001, ** p < 0.01, * p < 0.05, ns = not significant). (G) Representative images of LysoTracker staining in adult fat body of *WT, daw^[11]^/+, Tsc1^[12]^/+* and double mutants *daw^[11]^/+; Tsc1^[12]^/+*. Scale bar: 10 μm. Quantification shown on the right. N = 5. One-way ANOVA (*** p < 0.001, ** p < 0.01, * p < 0.05, ns = not significant).

Since we observed an induction of autophagy in *daw* knockdown flies, we next asked whether autophagy activity is required for daw-regulated cardiac aging. First, we fed control and *daw* knockdown flies with a lysosomal inhibitor chloroquine (CQ). As expected, CQ treatment increased cardiac arrhythmia in *daw* knockdown flies back to the similar levels seen in old wild-type flies (Figure 4A), while CQ treatment is nontoxic and did not lead to any mortality within two weeks of feeding (Figure S5D). To directly test whether autophagy plays any role in daw-regulated cardiac aging, we generated double knockdown flies by combining *UAS-daw* RNAi and *UAS-Atg1* RNAi fly lines. Atg1 is a serine/threonine-protein kinase that is essential for autophagy initiation, and has been previously shown to play a role in regulating cardiac function [32]. Cardiac-specific knockdown of *Atg1* increased both cardiac arrhythmia and diastolic intervals at young and old ages (Figure 4B), similar to the fly heart with activated babo (Figure 2D,E). Consistent with CQ treatment, cardiac-specific knockdown of *Atg1* attenuated cardioprotective effects of *daw* RNAi. Simultaneously knockdown of *Atg1* and *daw* in the heart led to an age-dependent increase in arrhythmia similar to control flies (Figure 4C). *Atg1* knockdown in *daw* RNAi background also slightly increased arrhythmia at young ages (Figure 4C). The knockdown efficiency of *daw* was not affected by combining *daw* RNAi and *Atg1* RNAi lines (Figure S5E). Thus, these results suggest that autophagic activity and Atg1 are required for daw-regulated cardiac aging.

TORC1 is a key negative regulator of autophagy (Figure S6A) [24]. We found that TORC1 activity, indicated by phosphorylation of Thor/EIF4EBP1, was reduced in *daw* knockdown flies (Figure S6B). We then tested whether daw could regulate autophagy and cardiac aging through TORC1 signaling. If TORC1 acts as a downstream effector of daw to regulate autophagy and cardiac aging, one would expect that activation of TORC1 can attenuate *daw* knockdown-mediated cardiac protection and autophagy induction. To test this possibility, we first generated double knockdown flies by combining *UAS-daw* RNAi and *UAS-Tsc1* RNAi. *Drosophila* Tsc1 (TSC complex subunit 1) is a negative regulator of TORC1, knockdown of *Tsc1* increased the phosphorylation of Thor/EIF4EBP1 in fly hearts (Figure S6B). The knockdown efficiency of *Tsc1* is shown in Figure S6C. Surprisingly, activation of TORC1 via *Tsc1* knockdown had no effects on cardiac arrhythmia (Figure 4D), but reduced diastolic intervals at young age (Figure 4E), which is very different from the cardiomyopathy resulted from cardiac-specific activation of INHB/activin receptor *babo* (Figure 2D,E). Furthermore, knockdown of *Tsc1* did not affect the cardioprotective role of *daw* silencing (Figure 4F). Simultaneously knockdown of *Tsc1* and *daw* showed similar arrhythmia index as *daw* RNAi alone. In addition, loss-of-function mutant *Tsc1^[12]^* did not rescue the elevated LysoTracker staining in *daw^[11]^* mutants (Figure 4G). These results suggest that activation of TORC1 is not required for daw-regulated autophagy and cardiac aging.

It is known that inhibition of TORC1 prolongs lifespan [15,33] and attenuates stress-induced cardiomyopathies [11,34 35–36]. However, the specific role of TORC1 in cardiac aging is not well understood. Here, we observed unique alternations of cardiac function in response to activation of TORC1 (via *Tsc1* knockdown), including decreased heart period (Tachycardia, fast heart rate), and reduced diastolic intervals (Figures 4E, S6D,E) These cardiac functional changes are not the same as age-induced cardiomyopathies, rather they resemble high-fat-diet-induced cardiac complications where TORC1 is known to play an important role [12]. Intriguingly, flies with cardiac-specific knockdown of *Tsc1* showed no age-dependent increases in heart period, diastolic intervals, and arrhythmia (Figure S6D–F). Similar cardiomyopathy phenotypes were also observed in other TORC1 activation mutants, such as 1) knockdown of *REPTOR* (Repressed by TOR), a recently identified bZIP transcription factor that is negatively regulated by TORC1 [37] (Figure S6G–I), and 2) overexpression of *Rheb* (Ras Homolog Enriched in Brain), a positive regulator of TORC1 (Figure S6J–L). Thus, it seems that INHB/activin-daw signaling and TORC1 exhibit different effects on cardiac function.

### daw genetically interacts with TORC2 (rictor) to control autophagy and cardiac aging

**Figure 5.**
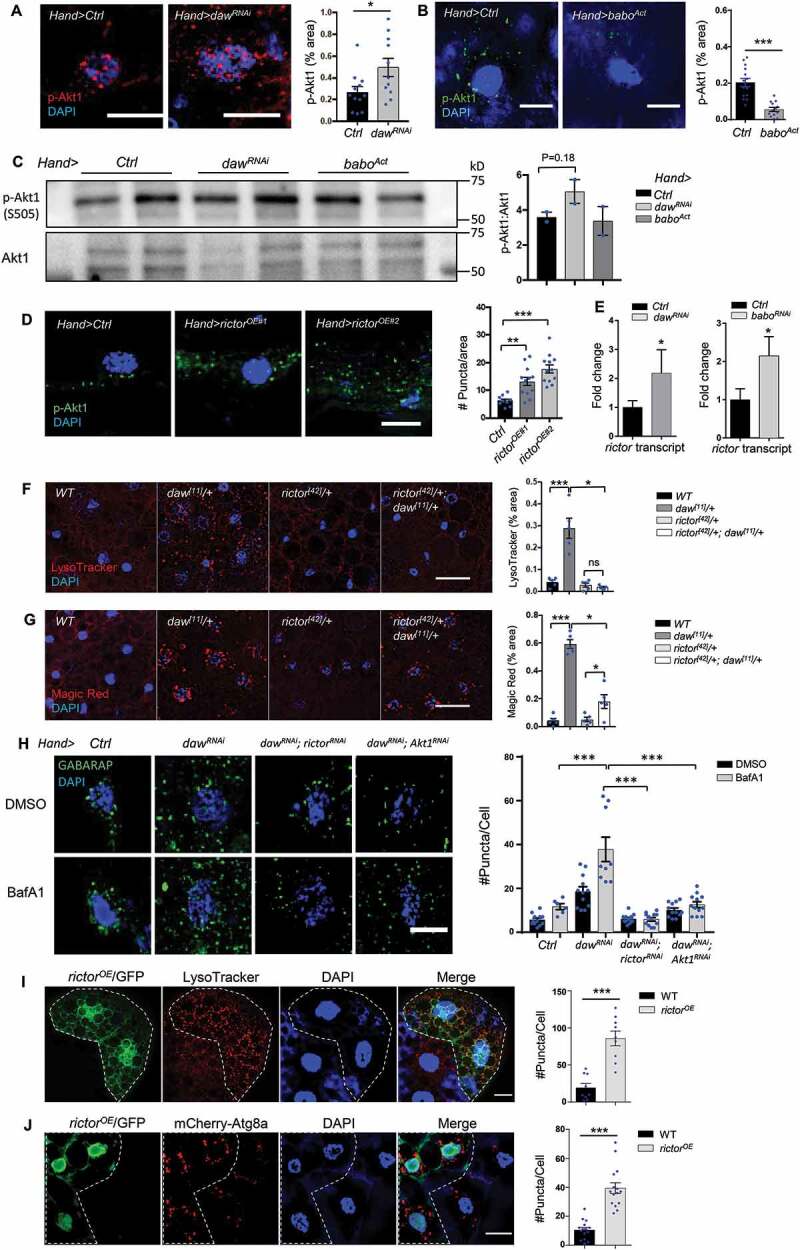
Daw genetically interacts with TORC2 (rictor) to control autophagy. (A) Representative images of p-Akt1 staining in cardiomyocytes of control and *daw* knockdown flies (*Hand-gal4*). Scale bar: 10 μm. Quantification shown on the right. N = 5. Student t-test (* p < 0.05). (B) Representative images of p-Akt1 staining in cardiomyocytes of control and *babo-*activated flies (*Hand-gal4*). Scale bar: 10 μm. Quantification shown on the right. N = 5. Student t-test (*** p < 0.001). (C) Western blot analysis on Akt1 phosphorylation of the hearts dissected from control, *daw* knockdown and *babo*-activated flies (*Hand-gal4*). Quantification of band intensity shown on the right. N = 3. Student t-test. (D) Representative images of p-Akt1 staining in cardiomyocytes of control and *rictor* overexpression (*Hand-gal4*). Two independent *rictor* overexpression used (#1: *UAS-rictor*, #2: *UAS-HA-rictor* [72]). Scale bar: 10 μm. Quantification shown on the right. N = 5. One-way ANOVA (*** p < 0.001, ** p < 0.01, * p < 0.05, ns = not significant). (E) QRT-PCR analysis of *rictor* expression in fly hearts with *daw* and *babo* knockdown (*Hand-gal4*). N = 3. Student t-test (* p < 0.05). (F) Representative images of LysoTracker staining in adult fat body of *WT, daw^[11]^/+, rictor^[42]^/+* and double mutants *rictor^[42]^/+; daw^[11]^/+*. Scale bar: 20 μm. Quantification shown on the right. N = 5. One-way ANOVA (*** p < 0.001, ** p < 0.01, * p < 0.05, ns = not significant). (G) Representative images of Magic Red staining in adult fat body of *WT, daw^[11]^/+, rictor^[42]^/+* and double mutants *rictor^[42]^/+; daw^[11]^/+*. Scale bar: 20 μm. Quantification shown on the right. N = 5. One-way ANOVA (*** p < 0.001, ** p < 0.01, * p < 0.05, ns = not significant). (H) Representative images of GABARAP immunostaining in cardiomyocytes of BafA1-treated control, *daw^RNAi^* alone, *daw^RNAi^; rictor^RNAi^*, and *daw^RNAi^; Akt1^RNAi^* flies. Scale bar: 20 μm. Quantification shown on the right. N = 15. Student t-test (*** p < 0.001). (I) Representative images of mosaic analysis of LysoTracker staining in larval fat body. Fat body clones were generated by crossing *rictor* overexpression lines into a FLPout line (*yw, hs-flp, UAS-CD8::GFP; Act>y+>Gal4,UAS-GFP.nls;UAS-Dcr-2*). Clones with *rictor* overexpression are GFP-positive cells (dashed lines). Scale bar: 20 μm. (J) Representative images of mosaic analysis of autophagosome staining in larval fat body. Fat body clones were generated by crossing *rictor* overexpression lines into a FLPout line carrying mCherry-Atg8a reporter (*hs-flp; endogenous P-3x mCherry-Atg8a, UAS-GFP/Cyo; Act>CD2> Gal4,UAS-Dcr-2*). Clones with *rictor* overexpression are GFP-positive cells (dashed lines). Scale bar: 20 μm.

Besides the regulation of p-Thor (Figure S6B), cardiac-specific knockdown of *daw* increased the phosphorylation of Akt1 (at Ser505) in the heart, the upstream regulator of TORC1 (Figure 5A). On the other hand, activated receptor babo decreased cardiac Akt1 phosphorylation (Figure 5B). It is interesting that the p-Akt1 appeared as fluorescence puncta in fly hearts. The p-Akt1 puncta might be associated with mitochondria, since it has been shown that MTORC2 and Akt1 are localized on mitochondria-associated endoplasmic reticulum (ER) membrane [38]. We further confirmed the regulation of Akt1 phosphorylation by TGFB-INHB/activin signaling using immunoblotting (Figure 5C). Although the activation of babo did not alter Akt1 phosphorylation in western blotting analysis, we think that the lack of changes in Akt1 phosphorylation might be due to the contamination of adjacent tissue (e.g., fat body). It is known that Akt1 can be activated and phosphorylated by two kinases at distinct positions, PDPK1/Pdk1 (3-phosphoinositide dependent protein kinase 1) at Thr308 and MTORC2 at Ser473 (Ser505 in flies) [39]. Cardiac-specific overexpressing *rictor* can also induced Akt1 phosphorylation (Figure 5D). Interestingly, knockdown of *daw* and *babo* upregulated the mRNA expression of *rictor* in fly hearts (Figure 5E). These results suggest that INHB/activin-daw is a negative regulator of MTORC2 (rictor).

Unlike TORC1, the role of TORC2 in the regulation of autophagy is unclear. Intriguingly, we found that loss-of-function mutants *rictor^[42]^* suppressed the high lysosomal activities in *daw^[11]^* mutants (Figure 5F,G). Similarly, mutations in *Sin1*, another core component of TORC2, blocked the elevated LysoTracker staining in *daw* mutants (Figure S7A,B). Cardiac-specific knockdown of either *rictor* or *Akt1* attenuated the induction of autophagosome formation by *daw* RNAi in fly hearts (Figure 5H). Furthermore, the mosaic analysis revealed that overexpression of *rictor* induced lysosome activity (Figure 5I) and increased the number of Atg8a-positive autophagosome in fat body (Figure 5J). Interestingly, unlike *Tsc1* mutants, *rictor^[42]^* did not suppress starvation-induced LysoTracker staining (Figure S7C). Similarly, activated TGFB/activin signaling (*babo^Act^*) did not block starvation-induced lysosome activity (Figure S7D). Taken together, these data suggest that TGFB-INHB/activin signaling and TORC2 (rictor) regulate autophagy differently from TORC1, and rictor acts downstream of TGFB-INHB/activin signaling in the regulation of autophagy.

**Figure 6.**
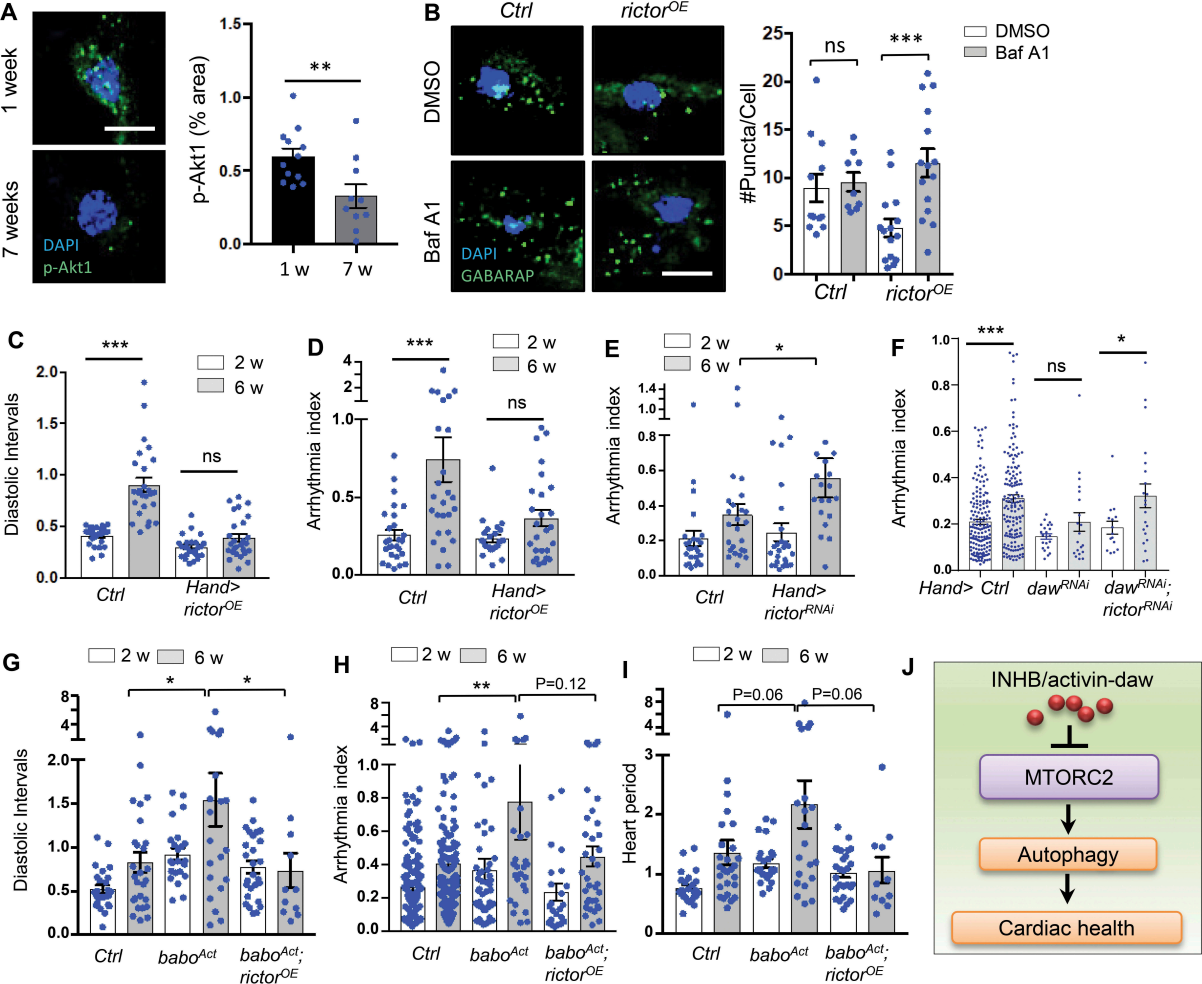
Heart-specific overexpression of *rictor* preserved cardiac function during aging. (A) Representative images of p-Akt1 staining in young and old cardiomyocytes of wild-type flies. Scale bar: 10 μm. Quantification shown on the right. N = 12. Student t-test (** p < 0.01). (B) Representative images of autophagic flux in control and *rictor* overexpressing cardiomyocytes at old ages (*Hand-gal4*). Scale bar: 10 μm. Quantification shown on the right. N = 15. One-way ANOVA (*** p < 0.001, ** p < 0.01, * p < 0.05, ns = not significant). (C-D) Diastolic intervals and arrhythmia index in flies with heart-specific overexpression of *rictor* (*Hand-gal4*). N = 19 ~ 26. One-way ANOVA (*** p < 0.001, ** p < 0.01, * p < 0.05, ns = not significant). (E) Cardiac arrhythmia index in flies with heart-specific knockdown of *rictor*. N = 24. One-way ANOVA (*** p < 0.001, ** p < 0.01, * p < 0.05, ns = not significant). (F) Cardiac arrhythmia index in flies with heart-specific knockdown of *daw* alone or *daw; rictor* double knockdown. N = 15 ~ 30. Student t-test (*** p < 0.001, * p < 0.05, ns = not significant). (G-I) Diastolic intervals, arrhythmia index, and heart period in flies with heart-specific expression of *babo^Act^*, or *babo^Act^; rictor^OE^* (*Hand-gal4*). N = 11 ~ 27. One-way ANOVA (*** p < 0.001, ** p < 0.01, * p < 0.05, ns = not significant). (J) Proposed model: INHB/activin-mediated inhibition of MTORC2 as a novel mechanism for age-dependent regulation of autophagy and cardiac health.

Lastly, we noticed that the levels of p-Akt1 decreased in aging hearts (Figure 6A), while overexpression of *rictor* induced autophagic flux in old fly hearts (Figure 6B). Given that rictor positively regulates autophagy, we predict that rictor is a cardiac protective factor. Indeed, cardiac-specific overexpression of *rictor* prevented age-related increases in diastolic intervals and arrhythmia (Figure 6C,D), while knockdown of *rictor* induced arrhythmia, especially at old ages (Figure 6E). Knockdown of *rictor* also attenuated the cardioprotection by *daw* RNAi (Figure 6F). Furthermore, overexpression of *rictor* attenuated the elevated diastolic intervals and arrhythmia in fly hearts with constitutively activated *babo* (Figure 6G–I), suggesting that fly TGFB/activin signaling regulates cardiac aging through TORC2 (rictor) (Figure 6J).

### Cardiac-specific reduction of INHB/activin-daw and overexpression of rictor prolong lifespan

**Figure 7.**
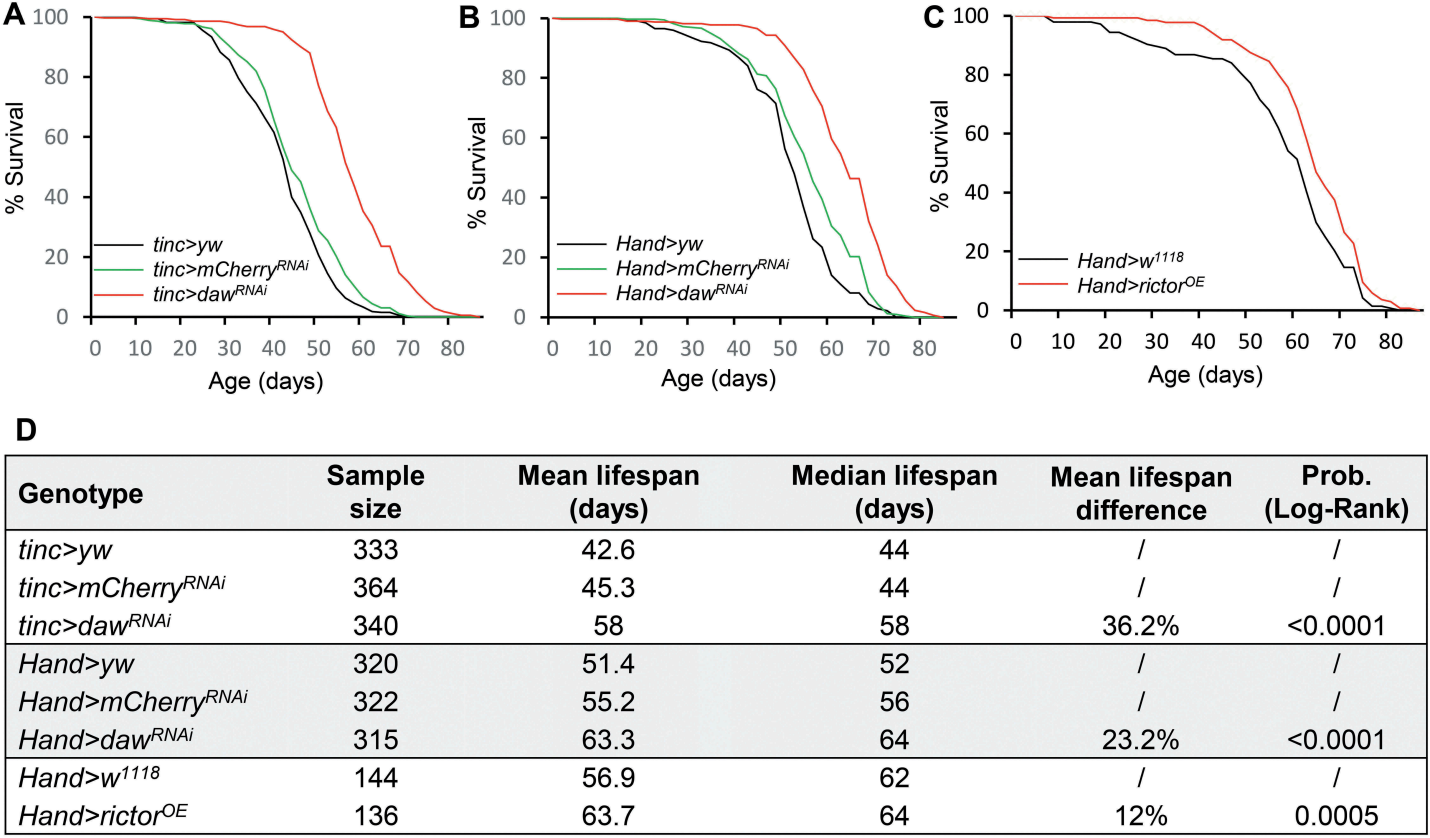
Cardiac-specific knockdown of *daw* and overexpression of *rictor* prolong lifespan. (A-B) Survival analysis of cardiac-specific (*tinc-gal4* and *Hand-gal4*) knockdown of *daw*. Two control lines used (*yw* and *mCherry* RNAi) were performed. Log-Rank test, p < 0.0001. (C) Survival analysis of heart-specific (*Hand-gal4*) overexpression of *rictor*. Log-Rank test, p = 0.0005. (D) Lifespan table to show sample size, mean and median lifespan of the survival analysis.

Tissue-specific hormonal signaling in systemic aging control has been previously reported [40 41–42]. However, whether prolonged organismal lifespan can be achieved by maintaining healthy hearts remains less clear. Here we tested the effects of cardiac-specific knockdown of INHB/activin signaling in longevity control. Knockdown of *daw* in the heart using two cardiac tissue drivers (*tinc-gal4* and *Hand-gal4*) significantly prolongs lifespan (36.2% and 23.2% extension of mean lifespan compared to control lines) (Figure 7A,B,D). Because daw is a hormonal factor, it remains to be determined whether daw regulates lifespan through cell-autonomous or non-autonomous mechanisms. Interestingly, cardiac-specific overexpression of *rictor* also extended lifespan (12% extension of mean lifespan) (Figure 7C,D). Together, our findings suggest that cardiac-specific INHB/activin-daw and TORC2 (rictor) signaling pathways play important roles in regulating autophagy, cardiac health, as well as longevity.

## Discussion

TGFB signaling plays vital roles in a wide range of human diseases, including cancer and cardiovascular diseases [43]. Originally identified as a reproductive hormone, INHB/activin, a TGFB subfamily member, has become an emerging target for the treatment of many human diseases [44]. In the present study, we discover a novel crosstalk between TGFB-INHB/activin and TORC2 in the regulation of autophagy and cardiac aging in *Drosophila*. Similar to our previous study in *Drosophila* flight muscle [20], reduction of TGFB-INHB/activin signaling alleviates age-related cardiac dysfunction, mainly through the regulation of cardiac autophagic activity. Mutant *daw* flies exhibit high autophagic flux and elevated lysosome activities. Importantly, we show that activation of TORC1 does not block *daw* knockdown-mediated cardioprotective effects. Instead, INHB/activin-daw signaling interacts with TORC2 subunit rictor to regulate autophagy and cardiac function. Activation of TORC2 alone can promote cardiac autophagy and maintain cardiac function at old ages. Taken together, our studies identify TGFB-INHB/activin signaling as a novel upstream regulator of TORC2, and INHB/activin-mediate inhibition of TORC2 might be a novel mechanism for age-related autophagy impairment and cardiomyopathy.

Although previous studies have linked TGFB family proteins to longevity regulation [20,21,45], the role of TGFB-INHB/activin signaling in cardiac aging is still not well understood. It has been shown that INHBA/ACTIVIN A serum levels significantly increase with age [46]. The mRNA expression of *INHBA/ACTIVIN A*, as well as serum INHBA/ACTIVIN A levels are positively correlated with heart failure [47,48], and hypertension in the elderly [49]. These findings suggest that activation of TGFB-INHB/activin signaling at old ages might be detrimental to the heart. It is known that INHBA/ACTIVIN A promotes cardiac fibrosis and myocardial damage after ischemia reperfusion [50]. Consistently, mice with heterozygous mutations of ACVR1B/ALK4 are protected from pressure overload-induced fibrosis and dysfunction [51,52]. Inhibition of TGFBR1/ALK5 also reduces myocardial infarction-induced systolic dysfunction and left ventricular remodeling in rats [53]. However, other studies suggest that INHBA/ACTIVIN A protects hearts from hypoxia/reoxygenation- and ischemia/reperfusion-induced cell death [54], even though it can promote cardiac apoptosis at higher concentrations [55]. The findings from our study support the detrimental effects of TGFB-INHB/activin signaling in cardiac function at old ages. This is consistent with the pro-aging action of TGFB and INHB/activin [20,45]. In addition, our results also suggest that daw-regulated cardiac aging through a Smox/SMAD2-independent pathway (Figure 1).

TGFB-INHB/activin signaling has been implicated in the regulation of a wide range of cellular processes, including cell death and proliferation, inflammation, fibrosis, and metabolic homeostasis [44,56 57–58]. The role of TGFB-INHB/activin signaling in the control of autophagy, a key tissue maintenance process, has only been carefully characterized recently [20,59,60]. In *Caenorhabditis elegans*, the reduction of INHB/activin-like protein DAF-7 suppresses beta-amyloid peptide-induced autophagosome accumulation [59]. Another recent study found that INHBA/ACTIVIN A blocked oxygen-glucose deprivation-induced autophagy through the inhibition of MAPK8/JNK and MAPK14/p38 MAPK pathways in neuronal PC12 cells [60]. Our previous studies show that knockdown of *Drosophila* INHB/activin-like protein daw in flight muscle induces autophagosome formation and autophagy gene expression [20]. However, how TGFB-INHB/activin signaling regulates autophagy and how this regulation contributes to tissue homeostasis during aging remain largely unknown. Our findings suggest that fly INHB/activin-like protein daw promotes cardiac aging through inhibition of autophagy and TORC2 signaling. Importantly, fly hearts with reduced *daw* expression maintain high levels of autophagosome turnover (or autophagic flux) throughout the life, whereas the autophagosome turnover is largely blocked in aged hearts of wild-type flies. The active autophagosome turnover at old ages is likely responsible for the healthy hearts seen in *daw* knockdown flies. Intriguingly, TORC2 but not TORC1 is required for daw-regulated cardiac autophagy. These findings reveal a novel mechanism for age-dependent autophagy regulation that is mediated through the interplay between INHB/activin and TORC2. On the other hand, our results further suggest that TGFB-INHB/activin acts as a key upstream regulator of TORC2 signaling.

The MTOR signaling, in particular, MTORC1, is a well-known autophagy regulator and it inhibits autophagosome formation by increasing the phosphorylation of the serine/threonine protein kinase Atg1 (ULK1/ULK2 in mammals), the initiator of the autophagy machinery [5]. Intriguingly, activation of TORC1 does not block the induction of autophagosome formation in *daw* knockdown flies, suggesting that there might be unknown TORC1-independent mechanisms involved in daw-regulated autophagy and cardiac aging. TORC1-independent autophagy regulation has been observed in several recent studies [61 62–63]. A genome-wide screen study identifies an important role of class III phosphatidylinositol 3-kinase in autophagy control under normal nutritional conditions [61]. The screen also revealed that growth factor signaling pathways, such as MAPK/ERK and AKT-FOXO3, negatively regulate autophagy by inhibiting the class III phosphatidylinositol 3-kinase cascade. Interestingly, we show that knockdown of *daw* leads to elevated Akt1 phosphorylation and increased expression of TORC2 subunit *rictor*, suggesting TORC2-AKT signaling may be involved in daw-regulated autophagy and cardiac aging. Indeed, mutation of *rictor* blocks the activation of autophagy in *daw* knockdown flies. MTORC2 is one of the two MTOR complexes that is insensitive to short-term rapamycin treatment, and is involved in glucose homeostasis and actin cytoskeleton reorganization [64,65]. The direct regulatory role of MTORC2 (RICTOR) in autophagy has not been previously established, although several studies suggest that mammalian RICTOR may be involved in autophagy activation under certain circumstances. For example, RICTOR is required for resveratrol-induced autophagy in rat myocardium and H9c2 cardiac myoblast cells [66]. In rat kidney NRK-52E cells, silencing *Rictor* blocks cisplatin-induced autophagy [67]. Our findings align with these studies suggesting that *Drosophila* rictor is a positive regulator of autophagy. However, conflicting findings are reported in other studies showing RICTOR negatively regulates autophagosome formation in mouse skeletal muscle [68], and LC3-II levels in human senescent endothelial cells [69]. Together, our results suggest that *Drosophila* TORC2 (rictor) is a positive regulator of autophagy, which is opposite to what TORC1 does. The distinct regulation of autophagy by 2 MTOR complexes may explain why MTORC1 and MTORC2 differentially control lifespan.

The positive regulation of autophagy by rictor identified in the present study suggests that activation of TORC2 may be cardioprotective. Indeed, we show that cardiac-specific overexpression of *rictor* slows cardiac aging, similar to *daw* knockdown. These findings are consistent with two recent studies showing that disruption of mammalian RICTOR induces cardiac dysfunction [13,14]. Cardiac-specific deletion of *Rictor* in mice induces dilation and decreases fractional shortening by activating MST1 kinase and hippo pathway [14]. Deletion of *Rictor* decreases lifespan in male mice [16], although conflicting results are reported in *C. elegans* [70,71]. Interestingly, we show that cardiac-specific overexpression of *rictor* prolongs *Drosophila* lifespan, which is consistent with shortened lifespan seen in *rictor* deletion mice [16]. Besides longevity regulation, it has been shown that *Drosophila* mutants of *rictor* and *Sin1* decrease the tolerance to heat stress [72] and overexpression of *rictor* rescues *Pink1* knockdown-induced mitochondrial aggregation in *Drosophila* indirect flight muscle [73]. Conversely, overexpression of PINK1 increases AKT phosphorylation in human SH-SY5Y neuroblastoma cells [74]. The rictor-Pink1 interaction suggests that rictor could be a potential positive regulator for mitophagy, a key process for the mitochondria quality control during aging. Interestingly, we found that p-Akt1 exhibits a puncta pattern in fly hearts. We speculate that the p-Akt1 puncta may be associated with mitochondria and ER subcompartment, since it has been shown that MTORC2 (RICTOR) is localized at the mitochondria-associated ER membrane [38]. Future work is needed to examine whether and how TORC2 (rictor) regulates mitophagy activity to maintain mitochondrial quality in the heart.

Different from TORC2, TORC1 is known to promote age-dependent increases in cardiac dysfunction in *Drosophila* [11,36]. Cardiac-specific overexpression of TORC1 increases stress-induced heart failure, whereas overexpression of *Tsc1* and *Tsc2* prevents the age-related increase of stress-induced heart failure [11]. Interestingly, we show that activation of TORC1 produced unique cardiac changes that are different from aging fly hearts. Cardiac-specific activation of *TORC1* via knockdown of *Tsc1* induced heart rate and decreased heart period, which is similar to cardiac dysfunction caused by high-fat-diet feeding [12]. A recent study found that MTORC1 activity does not increase with age in most tissues of wild-type mice, arguing that the assumption about increased MTOR signaling driving age-related pathology may be overly simplified [75]. Intriguingly, cardioprotective effects of MTOR were previously observed in a mouse model with cardiac-specific overexpression of MTOR (MTOR-Tg) [76]. The MTOR-Tg mice show attenuated inflammatory response and cardiomyopathy upon left ventricular pressure overload induced by transverse aortic constriction. It is unclear whether MTOR-Tg affects only MTORC1 or both MTOR complexes. Thus, the distinct role of MTORC1 and MTORC2 in stress- and aging-induced cardiac dysfunction remains to be further examined.

During normal aging, the heart undergoes complex phenotypic changes such as progressive myocardial remodeling, reduced myocardial contractile capacity, increased left ventricular wall thickness and chamber size, prolonged diastole as well as increased arrhythmia [3]. All of these biological changes can gradually alter cardiac functions and confer vulnerability of the heart to various cardiovascular stresses, thus increasing the chance of developing cardiovascular disease dramatically. The present study reveals an important role of TGFB-INHB/activin signaling pathway in the regulation of autophagy and cardiac aging in *Drosophila*. We also find an intriguing interaction between INHB/activin and TORC2 in the regulation of cardiac autophagy, as well as a new role of rictor in cardiac aging and longevity control in *Drosophila*. It remains to be determined how INHB/activin regulates TORC2 (rictor) in fly hearts. It is possible that TGFB-INHB/activin signaling regulates rictor through both transcriptional activation (e.g., INS-FOXO signaling [77]) and post-translational modification. Additionally, cardiac-specific *daw* knockdown and *rictor* overexpression extend fly lifespan, suggesting that longevity can be achieved by maintaining a healthy heart. Our findings also suggest that daw itself or other unidentified hormonal factors secreted from cardiomyocytes may play an important role in systemic aging control. Hence, it remains to be determined the mechanistic basis for INHB/activin-TORC2-mediated control of cardiac aging and longevity, which will eventually help the development of therapeutic interventions targeting INHB/activin and TORC2 for the treatment of age-related cardiovascular diseases.

## Materials and methods

### Fly husbandry and stocks

Flies were maintained at 25°C, 60% relative humidity and 12 h light/dark (40% relative humidity was used in the experiments from Figures 1 and 2, which were performed in Bodmer laboratory). Adults were reared on agar-based diet with 0.8% cornmeal, 10% sugar, and 2.5% yeast (unless otherwise noted). Fly stocks used in the present study are: *UAS-daw* RNAi (Bloomington *Drosophila* Stock Center [BDSC], 34974, BDSC, 50911, and Vienna Drosophila Resource Center [105309]), *UAS-daw* (*daw^OE^*, Gift from Michael O’Connor, University of Minnesota Twin Cities), *UAS-Smox* RNAi (BDSC, 26756), *UAS-babo* RNAi (BDSC, 25933, 40866), *UAS-babo^Act^/babo^Q302D^* [78], *UAS-Tsc1* RNAi (BDSC, 52931, 54034), *UAS-REPTOR* RNAi (BDSC, 25983), *UAS-Rheb* (BDSC, 9688), *UAS-Atg1* RNAi (BDSC, 26731), *UAS-rictor* RNAi (BDSC, 36699), *daw^[11]^* [79], *Tsc1^[12]^* [80], *rictor^[42]^* [81], *Sin1^[e03756]^* (BDSC, 18188), *UAS-rictor* [72], *UAS-HA-rictor* [72], *UAS-Akt1* RNAi (BDSC, 31701), *Hand/Hand4.2-gal4* [82], and *tinc/tinc∆4-gal4* [83], *Ugt36A1/Dot-gal4* [84], *Hand-GS-gal4* [85], *UAS-mCherry-Atg8a* (BDSC, 37750), *Atg8a^∆4^* [86], *UAS-GFP.nls* (BDSC, 4775), *fln/IFM-gal4, da-GS-gal4* [87], *UAS-mCherry-GFP-Atg8a* (Gift from Gábor Juhász, Eotvos Lorand University, *r4-gal4* (Gift from Marc Tatar, Brown University).

*UAS-daw* RNAi (BDSC, 34974) and *UAS-babo* RNAi (BDSC, 25933) were backcrossed into *yw^R^* background for 5 ~ 7 generations, and *yw^R^* flies were used as control or wild-type (WT) flies in most of the experiments. For other UAS-RNAi lines that were not backcrossed to *yw^R^*, following genotypes were used as control: y^1^ sc* v^1^; P[VALIUM20-mCherry]attP2 (BDSC, 35785), *y^1^ v^1^; P[CaryP]attP2* (BDSC, 36303), or *y^1^ v^1^; P[CaryP]attP40* (BDSC, 36304). Female flies were used in all experiments. RU486 (Mifepristone; Cayman Chemical, 84371-65-3) were used to activate *Hand-GS-Gal4* at a final concentration of 200 µM mixed in food.

### Mosaic analysis

Two FLPout lines were used: 1). *hs-flp; endogenous P-3x mCherry-Atg8a, UAS-GFP/Cyo; Act>CD2> Gal4,UAS-Dcr-2/TM6*) (kindly provided by Gábor Juhász, Eotvos Lorand University). 2). *yw, hs-flp, UAS-CD8::GFP; Act>y+>Gal4,UAS-GFP.nls;UAS-Dcr-2/SM6::TM6B* (kindly provided by Jun-yuan Ji, Texas A&M University, originally generated by Bruce Edgar, University of Utah). To generate clones, freshly laid eggs (within 4–6 h) were heated in a 37°C water bath for 45 min. Early L3 larvae (84 h after egg laying) were used in the experiments.

### Fly heartbeat analysis

To measure cardiac function parameters, semi-intact *Drosophila* adult fly hearts were prepared according to previously described protocols [22]. High-speed 3000 frames movies were taken at a rate of 100 frames per second using a Hamamatsu ORCA-Flash4.0 digital CMOS camera (Hamamatsu Photonics) on an Olympus BX51WI microscope with a 10X water immersion lens. Hamamatsu EM-CCD 9300 camera was used in the experiments from Figures 1 and 2, which were performed in Bodmer laboratory. The live images that contain heart tub within abdominal A3 segment were processed using HCI imaging software (Hamamatsu Photonics). M-modes and cardiac parameters were generated using SOHA, a MATLAB-based image application as described previously [22]. The M-mode provides a snapshot of the movement of heart wall over time. Cardiac parameters were calculated as below: Diastolic interval (DI) is the duration for each heart relaxation phase (diastole). Heart period (HP) is the pause time between the two consecutive diastoles (HP is the reciprocal of heart rate, HR). Systolic interval (SI) was calculated as the HP minus the DI. Arrhythmia index (AI) is the standard deviation of all HP in each fly normalized to the median HP. Cardiac output was calculated using following equation: (π r[d]^2^ – π r[s]^2^) x HR. r(d) is the radius of the heart tube at diastolic phase, while r(s) is the radius of the heart tube at systolic phase. Fractional shortening was calculated as (diastolic diameters – systolic diameters)/diastolic diameters.

### Quantitative RT-PCR

RNA extraction and cDNA synthesis were performed using a Cells-to-CT kit (Thermo Fisher Scientific, 44-029-54) from ~15 dissected adult hearts. QRT-PCR was performed with a Quantstudio 3 Real-Time PCR System (Thermo Fisher Scientific). mRNA abundance of each gene was normalized to the expression of *RpL32* (Ribosomal protein L32) by the method of comparative C_T_. Primer sequences are listed in Table S1.

### Antibodies and immunostaining

Since commercial antibodies for *Drosophila* Atg8a show nonspecific staining in immunostaining of fly tissues (data not shown), we used a human GABARAP (E1J4E) antibody (1:300) (Cell Signaling Technology [CST], 13733) to stain and detect endogenous *Drosophila* Atg8a. The epitope used to generate GABARAP antibody has identical amino acid sequences to *Drosophila* Atg8a protein (personal communication with Cell Signaling Technology and see sequence alignment in Figure S2A). This antibody has been previously used in several *Drosophila* autophagy studies [25–27], and verified by Kim et al. [28]. We further verified the specificity of the GABARAP antibody using an Atg8a loss-of-function mutant *Atg8a^∆4^* (Figure S2B–D). Other antibodies used for immunostaining are list below: ref(2)P (1:1000) (previously generated by the Nezis laboratory) [31], p-Smox/SMAD2 antibody (1:500; CST, 3108), p-EIF4EBP1 (1:300; CST, 2855), p-AKT1 (Ser473, 1:300; CST, 4060), Alexa Fluor 488-conjugated phalloidin (or Alexa Fluor 594) for F-actin staining (Thermo Fisher Scientific, A12379, A12381). All fluorescence-conjugated secondary antibodies were from Jackson ImmunoResearch (Alexa Fluor 488, 711-545-152; Alexa Fluor 594, 711-585-152).

For immunostaining, adult female flies were collected and dissected in PBS (Fisher Scientific, AAJ61196AP). Hearts were fixed in 4% paraformaldehyde for 15 min at room temperature (RT). After washing in PBS with 0.1% Triton X-100 (Fisher Scientific, BP151-100) (PBST), the fixed hearted were blocked in 5% normal goat serum (NGS; Jackson ImmunoResearch, 005-000-121) diluted in PBST for 1 h at RT. Hearts were then washed with PBST and incubated overnight at 4°C with primary antibodies diluted in 5% NGS. After washing with PBST, the samples were incubated for 2 h at RT with appropriate fluorescence-conjugated secondary antibodies. Hearts were mounted in ProLong Gold anti-fade reagent (Thermo Fisher Scientific, P36930) before being imaged using an epifluorescence-equipped Olympus BX51WI microscope.

### Imaging analysis and quantification

For imaging analysis and quantification, fluorescence images were first processed using the deconvolution module in Olympus cellSens Dimensions software, and then the number of puncta or fluorescent area/intensity in a selected region of interest (ROI, ~707 µm^2^) surrounding cardiomyocyte nuclei were measured with the “Measure and Count” module in Olympus cellSens software. To quantify autophagosome in fat body, the number of puncta per cell was measured. The imaging quantifications were done single- or double-blind.

### LysoTracker and Magic Red staining

Acidic organelles (including autolysosome) were monitored by staining tissue with 100 nM LysoTracker Red DND-99 (Thermo Fisher Scientific, L7528) for 5 min at room temperature. Lysosomal CtsB1 (cathepsin B1) activities were monitored using Magic Red Cathepsin-B Assay kit (ImmunoChemistry Technologies, 938) following the manufacturer’s manual. Nuclei were stained with either DAPI or Hoechst 33342 (1 µg/ml) (ImmunoChemistry Technologies, 938).

### Western blotting for Atg8a lipidation and Akt1 phosphorylation

Antibodies for western blot included: ACTB (actin beta) antibody (1:2000) (CST, 4967), GABARAP (E1J4E) antibody (1:2000) (CST, 13733), *Drosophila* p-Akt1 (Ser505) (1:1000) (CST, 4054), AKT1 (Pan) (1:2000) (CST, 4691), and HRP-conjugated secondary antibodies (Jackson ImmunoResearch, 711-035-152). For Atg8a lipidation, flies were first transferred to centrifuge tubes containing 1x laemmli buffer (Bio-Rad Laboratories, 1610737) (10 µl buffer for each milligram of fly), and heated at 100°C for 3 min. The samples were then homogenized and heated again at 100°C for 3 min. After the centrifuge at 14000 x g for 5 min, supernatants were collected and loaded onto Mini-PROTEAN precast gels (Bio-Rad Laboratories, 456–1095). Following incubation with primary and secondary antibodies, the blots were visualized with Pierce ECL Western Blotting Substrate (Thermo Fisher Scientific, PI34577). For Akt1 phosphorylation, RIPA lysis buffer (Thermo Fisher Scientific, PI36978) was used to extract protein samples.

### Bafilomycin A1 and chloroquine treatment

For bafilomycin A_1_ treatment, semi-intact hearts were incubated with 100 nM of bafilomycin A_1_ (Fisher Scientific, AAJ61835MCR) in artificial hemolymph (buffer receipt in [22]) for 2 h at room temperature prior to the appropriate immunostaining. DMSO was used as a control.

For chloroquine treatments, 100 µl of 20 mM chloroquine diphosphate salt, CQ (Fisher Scientific, ICN19391925) was added onto the fly food. Flies were fed with chloroquine for at least 24 h prior to the cardiac analysis or the western blots. To test the toxicity of CQ treatment, we performed a survival analysis by feeding *yw^R^* flies with 20 mM CQ and observed the mortality within a period of within two weeks (about 100 flies per treatment). Flies were transferred to fresh CQ food every day and fly mortality was recorded daily.

### Demography and survival analysis

Newly enclosed female flies were allowed to mate for two days, then separated from males and assigned to replicate one-liter demography cages at a density of 100–125 flies per cage. Three independent cages were set-up per genotype. Food was changed every 2 d, at which time dead flies were removed from the cage and counted. Survival analysis was conducted with JMP statistical software (SAS Institute), and data from replicate cages were combined. Mortality distributions were compared by Log-rank test.

### Statistical analysis

GraphPad Prism (GraphPad Software) was used for statistical analysis. To compare the mean value of treatment groups versus that of control, either student t-test or one-way ANOVA was performed using Tukey multiple comparison. The effects of mutants during aging were analyzed by two-way ANOVA, including Tukey multiple comparisons test. In SOHA analysis, the outliers were identified using Robust regression and Outlier removal (ROUT) method (Q = 1%) prior to the data analysis.

## Supplementary Material

Supplemental Material
