# Supplementary material for: TGFB-INHB/activin signaling regulates age-dependent autophagy and cardiac health through inhibition of MTORC2
Source: Autophagy. 2019 Dec 29;16(10):1807–22. doi: 10.1080/15548627.2019.1704117 (PMC8386626; doi:10.1080/15548627.2019.1704117)
Supplement: Supplemental Material [file KAUP_A_1704117_SM1783.docx]

**Supporting Information**

**TGFB-INHB/activin signaling regulates age-dependent autophagy and cardiac health through inhibition of MTORC2**

Kai Chang^1§^, Ping Kang^1§^, Ying Liu^1^, Kerui Huang^1^, Ting Miao^1^, Antonia P. Sagona^3^, Ioannis P. Nezis^3^, Rolf Bodmer^2^, Karen Ocorr^2^, Hua Bai^1^*

1 Department of Genetics, Development, and Cell Biology, Iowa State University, Ames, IA 50011, USA

2 Development, Aging, and Regeneration Program, Sanford-Burnham-Prebys Medical Discovery Institute, La Jolla, California 92037, USA

3 School of Life Sciences, University of Warwick, Coventry CV4 7AL, UK

***Corresponding Author:**

Hua Bai

Email: hbai@iastate.edu

**§ These authors contribute equally to this work.**

**This file includes:**

Supplementary Figures

Supplementary Table S1

**Supplemental Figures**

**

**

**Figure S1.** Validation of cardiac drivers and RNAi lines. (**A**) Representative images of two cardiac driver lines crossing into *UAS-GFP.nls*. Heart tube is located between two dashed lines. PC: pericardial cells. Scale bar: 40 μm. (**B**) Quantification of GFP intensity in Panel **S1A**. N=4. Student t-test (*** p<0.001, ** p<0.01, * p<0.05, ns = not significant). (**C-D**) QRT-PCR analysis on the knockdown efficiency of three *daw* RNAi lines (*Da-GS-gal4* and *IFM-gal4* used). N=3. Student t-test (* p<0.05). (**E-H**) Age-related changes in systolic intervals, fractional shortening, diastolic diameter, and systolic diameter in control (*Ctrl*) and cardiac-specific *daw* knockdown flies (*daw^RNAi^*) (*Hand-gal4* used). N=7~20. One-way ANOVA followed by Tukey multiple comparisons test (* p<0.05, ** p<0.01, *** p<0.001, ns = not significant). (**I-J**) Age-related changes in arrhythmia index of three control fly lines. N=10~34. Student t-test (* p<0.05, ** p<0.01, *** p<0.001, ns = not significant). (**K**) Age-related changes in diastolic interval in control (*Ctrl*) and cardiac-specific *daw* overexpression flies (*daw^OE^*) (*Hand-gal4* used). N=7~20. Student t-test (* p<0.05). (**L**) Age-related changes in cardiac output in control (*Ctrl*) and cardiac-specific *babo* knockdown flies (*babo^RNAi^*) (*tinc-gal4* used). N=15~30. One-way ANOVA followed by Tukey multiple comparisons test (* p<0.05, ** p<0.01, *** p<0.001, ns = not significant). (**M-N**) Age-related changes in diastolic diameter, and systolic diameter in control (*Ctrl*) and cardiac-specific *babo* knockdown flies (*babo^RNAi^*) (*tinc-gal4* used). N=15~30. Student t-test (ns = not significant).

**
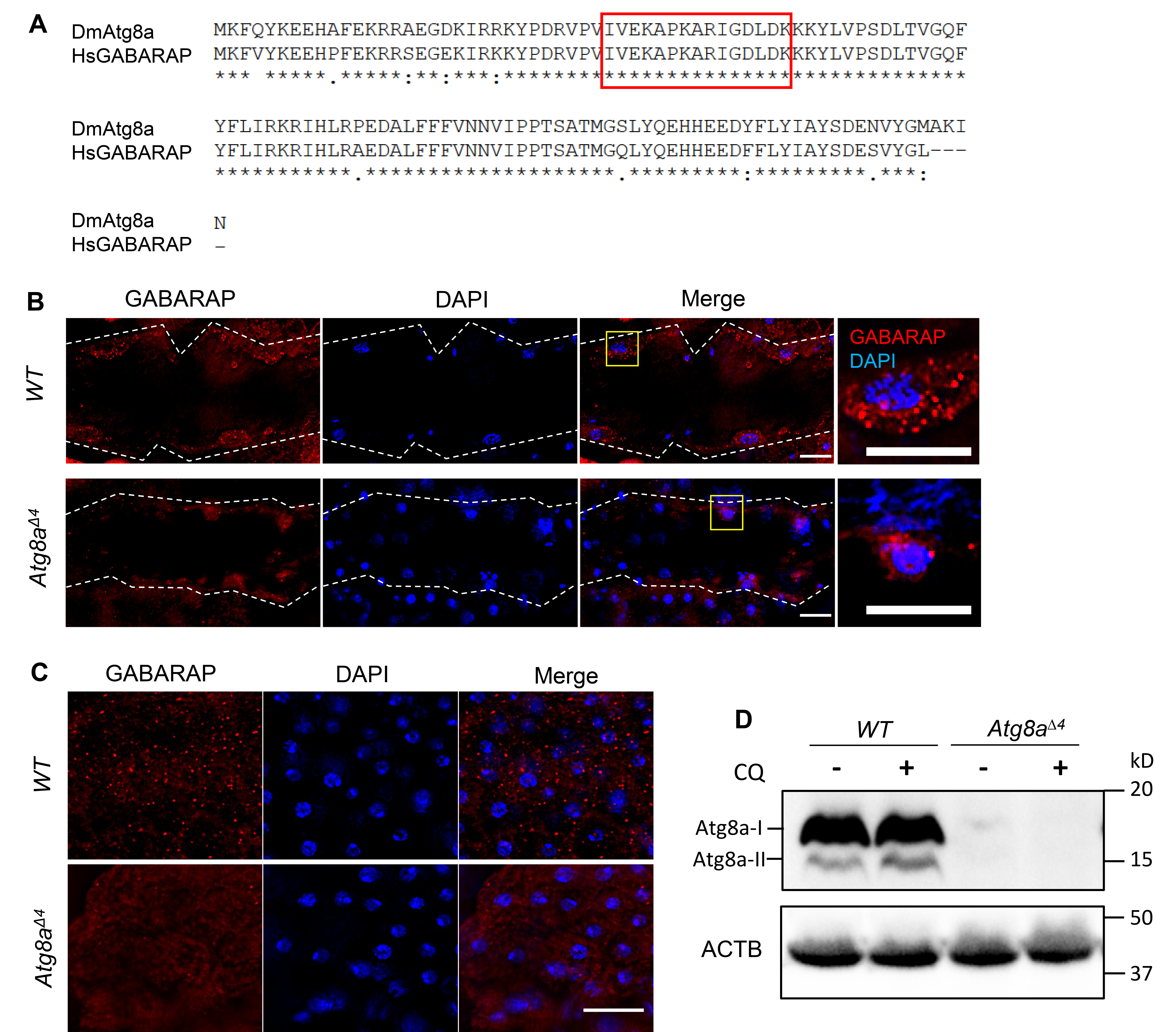
**

**Figure S2.** Validation of GABARAP antibody. (**A**) Multiple sequence alignment between *Drosophila* Atg8a and human GABARAP using ClustalW (<https://www.genome.jp/tools-bin/clustalw>). The red box indicates the predicted epitope sequences used in producing GABARAP antibody. (**B**) Representative images of GABARAP immunostaining in fly hearts of *WT* and *Atg8a^∆4^* mutants. Heart tube is located between 2 white dashed lines. The panels on the right are the zoomed-in images. Scale bar: 20 μm. (**C**) Representative images of GABARAP immunostaining in adult fat body of *WT* and *Atg8a^∆4^* mutants. The panels on the right are the zoomed-in images. Scale bar: 20 μm. (**D**) Western blots testing GABARAP antibody using *Atg8a^∆4^* mutants.

**
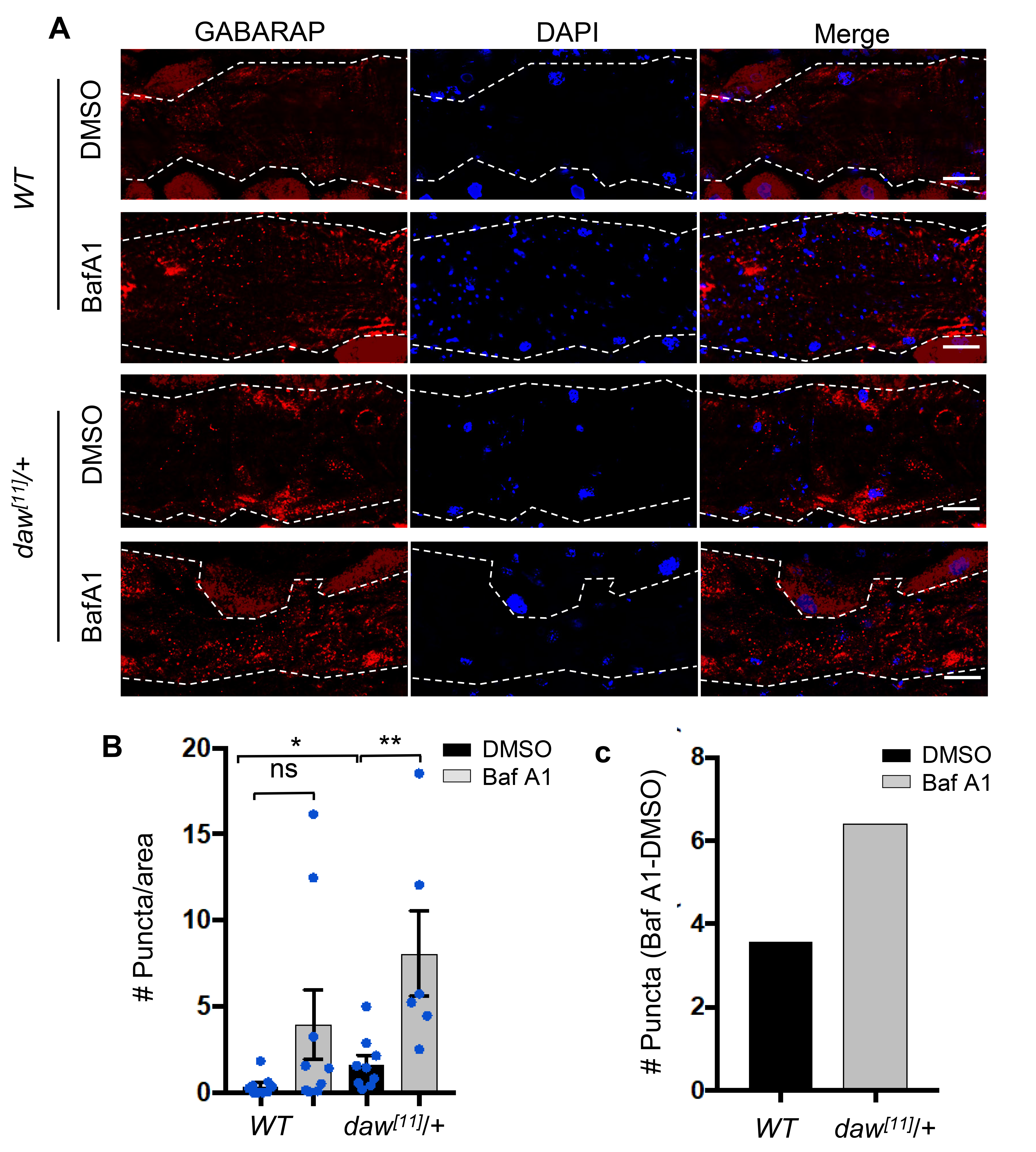
**

**Figure S3.** daw inhibits autophagy flux in the heart. (**A**) Representative images corresponding to anti-HsGABARAP immunostaining in fly hearts of *WT* and *daw^[11]^/+* mutants treated with or without BafA1. Heart tube is located between 2 white dashed lines. Scale bar: 20 μm. (**B**) Quantification of Panel **S3A**. N=5. One-way ANOVA (*** p<0.001, ** p<0.01, * p<0.05, ns = not significant). (**C**) Quantification of BafA1-induced puncta between *WT* and *daw^[11]^/+* mutants.

**

**

**Figure S4.** Autophagy flux decreases in aging hearts. (**A**) Representative images of GABARAP immunostaining in young and old fly hearts of *WT* treated with or without BafA1. Heart tube is located between 2 white dashed lines. Scale bar: 20 μm. The panels on the right are the zoomed-in images (Scale bar: 10 μm). (**B**) Quantification of Panel **S4A**. N=15. One-way ANOVA (*** p<0.001, ** p<0.01, * p<0.05, ns = not significant). (**C**) Representative images showing mCherry-GFP-Atg8a tandem reporter in wild-type fly hearts treated with or without BafA1. Heart tube is located between two dashed lines. Scale bar: 20 μm. (**D**) Representative images showing mCherry-GFP-Atg8a tandem reporter in wild-type 3^rd^ instar larval fat body treated with or without BafA1. Scale bar: 20 μm.

**
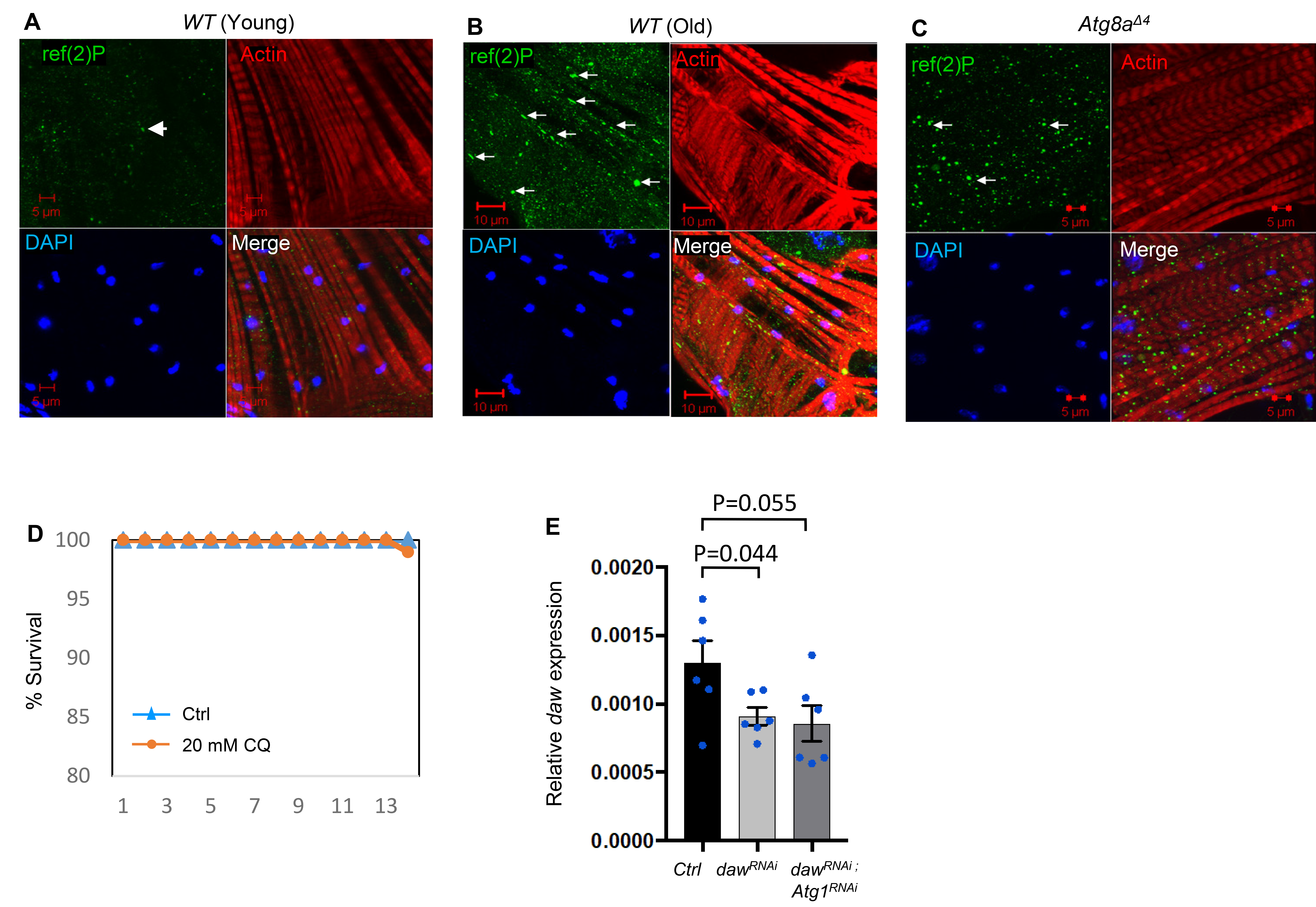
**

**Figure S5.** Aging increases the accumulation of ref(2)P in the heart. (**A-B**) Representative images of ref(2)P in young and old hearts. Scale bar: 10 μm. (**C**) Representative images of ref(2)P in the heart of *Atg8a^∆4^* mutants. Scale bar: 10 μm. (**D**) Survival curve of CQ-treated flies. Wild-type flies (*yw^R^*) were fed on 20 mM CQ (about 100 flies per treatment). (**E**) QRT-PCR analysis on the knockdown efficiency of *daw^RNAi^*, or *daw^RNAi^;Atg1^RNAi^* combine lines. N=3. Student t-test.





**Figure S6.** Activation of TORC1 alters heart period, but not arrhythmia. (**A**) Schematic diagram for the PI3K-AKT-TOR signaling pathway. (**B**) Representative images of p-Thor staining in cardiomyocytes of *daw* and *Tsc1* RNAi flies (*tinc-gal4* used). Scale bar: 10 μm. Quantification shown on the right. N=6. Student t-test (** p<0.01). (**C**) QRT-PCR analysis of the knockdown efficiency of *Tsc1* RNAi. Student t-test (*** p<0.001). (**D-F**) Heart period, diastolic intervals, and arrhythmia in control (*Ctrl*) and cardiomyocyte-specific *Tsc1* knockdown flies (*tinc-gal4*). N=14~18. One-way ANOVA (*** p<0.001, ** p<0.01, * p<0.05, ns = not significant). (**G-I**) Heart period, diastolic intervals, and arrhythmia in control (*Ctrl*) and cardiomyocyte-specific *REPTOR* knockdown flies (*tinc-gal4*). N=13~28. One-way ANOVA (*** p<0.001, ** p<0.01, * p<0.05, ns = not significant). (**J-L**) Heart period, diastolic intervals, and arrhythmia in control (*Ctrl*) and flies overexpressing *Rheb* (*tinc-gal4*). N=21~31. One-way ANOVA (*** p<0.001, ** p<0.01, * p<0.05, ns = not significant).

**
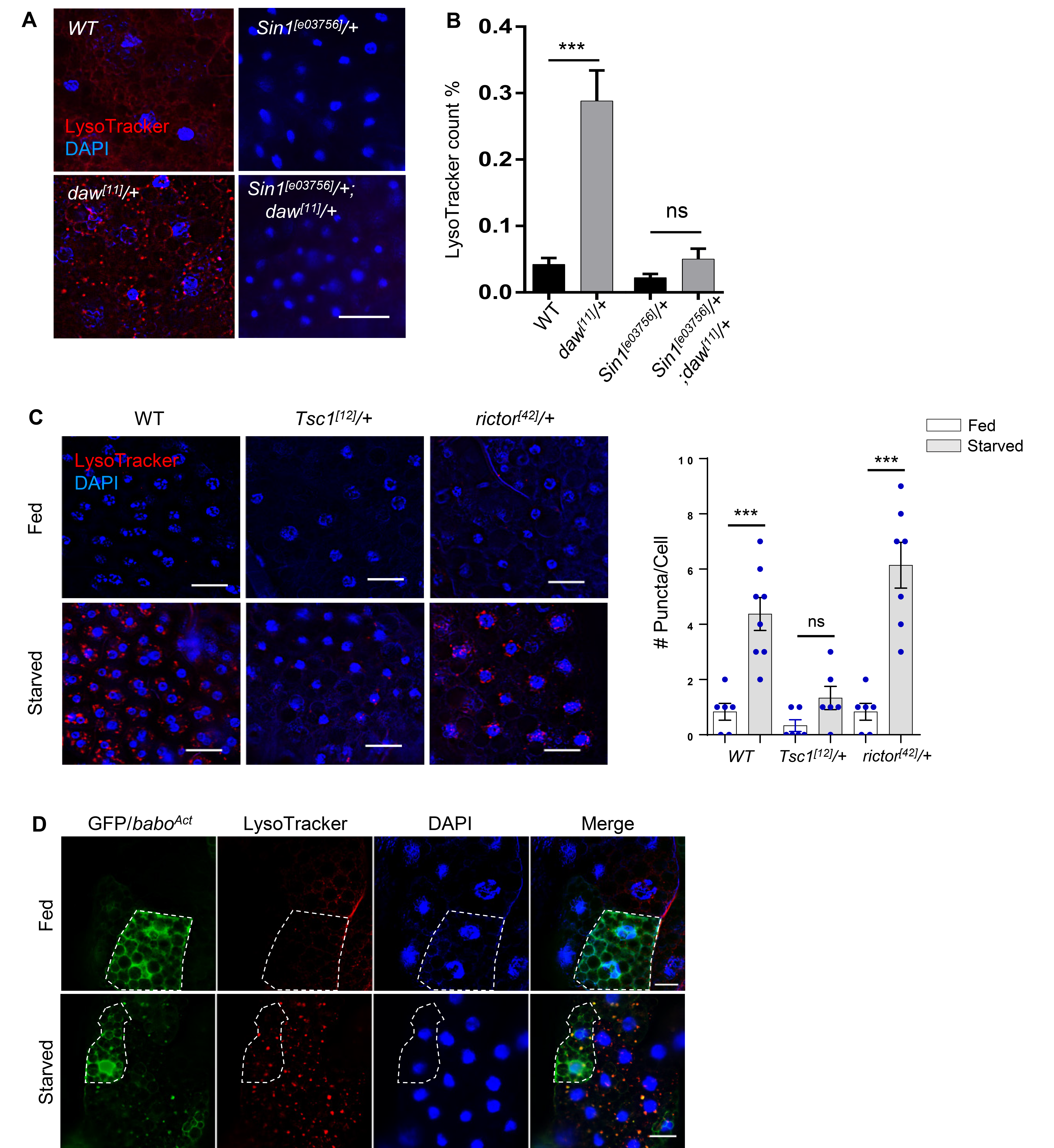
**

**Figure S7.** Autophagy regulation by mTORC2. (**A**) Representative images of LysoTracker staining in adult fat body of *WT*, *daw^[11]^/+*, *Sin1^[e03756]^/+* and double mutant *Sin1^[e03756]^/+*; *daw^[11]^/+*. Scale bar: 20 μm. (**B**) Image quantification of Panel **S7A**. N=5. One-way ANOVA (*** p<0.001, ** p<0.01, * p<0.05, ns = not significant). (**C**) Representative images of LysoTracker staining in fed and starved WT, *Tsc1^[12]^/+* mutants, and *rictor^[42]^/+* mutants. Scale bar: 20 μm. Image quantification shown on the right. N=5~8. Student t-test (** p<0.01, ns = not significant). (**D**) Representative images of mosaic analysis on LysoTracker staining in larval fat body of *babo^Act^* flies upon starvation. Larval fat body clones were generated using a FLPout line (*yw, hs-flp, UAS-CD8::GFP; Act>y+>Gal4,UAS-GFP.nls;UAS-Dcr2*). Clones with *babo^Act^* expression are GFP-positive cells (dashed lines). Scale bar: 20 μm.


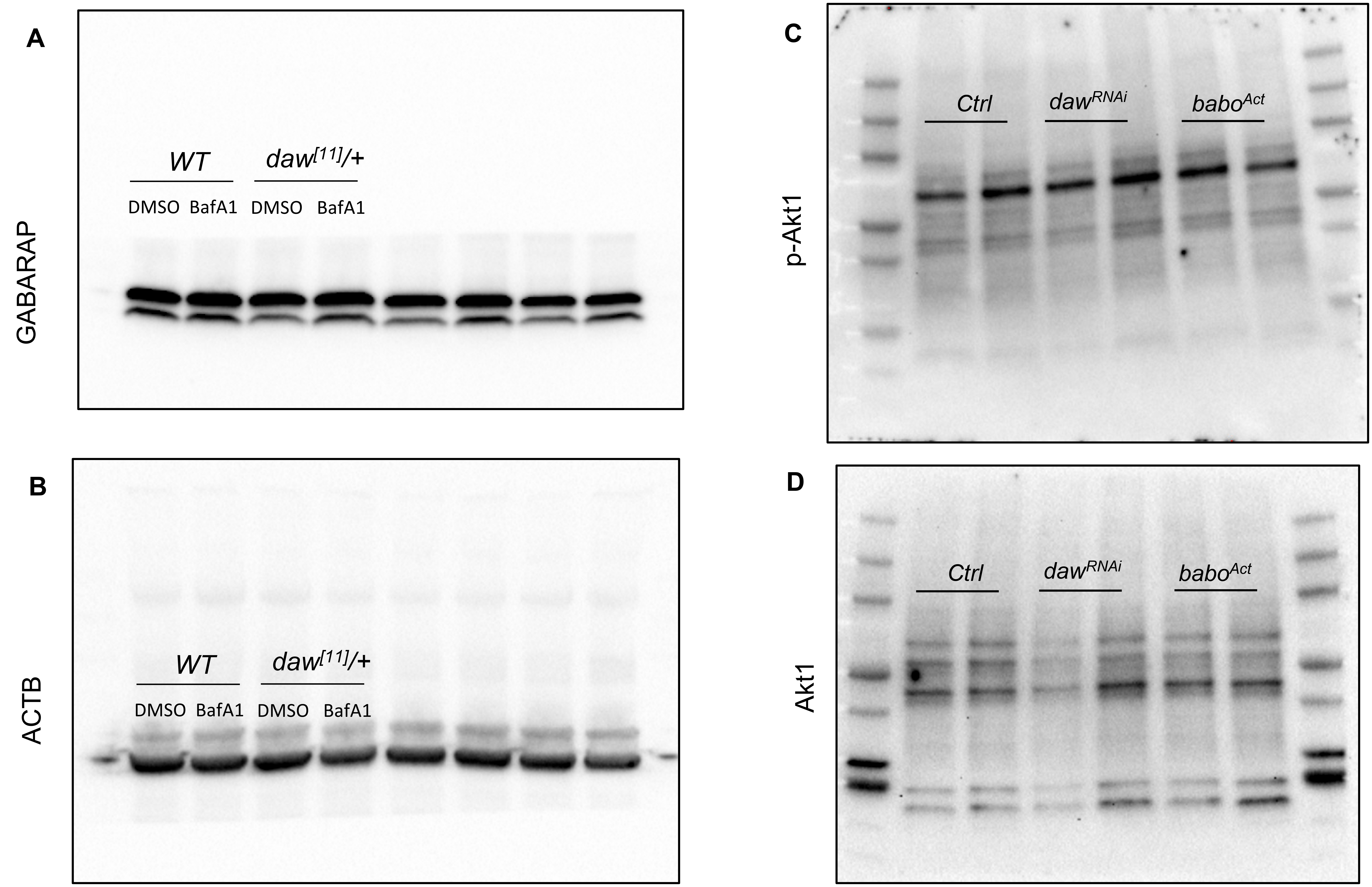


**Figure S8.** Original western blot images. (**A**) Original western blot of Fig. 3b (anti-GABARAP). (**B**) Original western blot of Fig. 3b (anti-ACTB). (**C**) Original western blot of Fig. 5c (anti-p-AKT1). (**D** Original western blot of Fig. 5c (anti-AKT1).

**Table S1. Primer list**

| Primer name | Primer sequence (5’-3’) | Symbol | FlyBase ID |
| --- | --- | --- | --- |
| RpL32-f | AAGAAGCGCACCAAGCACTTCATC | RpL32 | FBgn0002626 |
| RpL32-r | TCTGTTGTCGATACCCTTGGGCTT | RpL32 | FBgn0002626 |
| daw-f | TGAGCCACCTCATCCAAATCACCT | daw | FBgn0031461 |
| daw-r | TCGATCACGATGAATGGCCGGTAA | daw | FBgn0031461 |
| rictor-f | CCCAACTGGCTAGCGTTTAT | rictor | FBgn0031006 |
| rictor-r | GGCCTTTGCTTTCGTTTGAG | rictor | FBgn0031006 |
| Tsc1-f | TCGATTGGCTGGCGATAACA | Tsc1 | FBgn0026317 |
| Tsc1-r | GGTCGCTGTGAACTCTGCTA | Tsc1 | FBgn0026317 |
